# Thermal management of chips by a device prototype using synergistic effects of 3-D heat-conductive network and electrocaloric refrigeration

**DOI:** 10.1038/s41467-022-33596-z

**Published:** 2022-10-04

**Authors:** Ming-Ding Li, Xiao-Quan Shen, Xin Chen, Jia-Ming Gan, Fang Wang, Jian Li, Xiao-Liang Wang, Qun-Dong Shen

**Affiliations:** 1 Department of Polymer Science and Engineering, MOE Key Laboratory of High Performance Polymer Materials and Technology, School of Chemistry and Chemical Engineering, Nanjing University, Nanjing, 210023 China; 2 Materials Research Institute, The Pennsylvania State University, University Park, Pennsylvania 16802 USA; 3 State Key Laboratory of Analytical Chemistry for Life Science, School of Chemistry and Chemical Engineering, Nanjing University, Nanjing, 210023 China

**Keywords:** Ferroelectrics and multiferroics, Polymers

## Abstract

With speeding up development of 5 G chips, high-efficient thermal structure and precise management of tremendous heat becomes a substantial challenge to the power-hungry electronics. Here, we demonstrate an interpenetrating architecture of electrocaloric polymer with highly thermally conductive pathways that achieves a 240% increase in the electrocaloric performance and a 300% enhancement in the thermal conductivity of the polymer. A scaled-up version of the device prototype for a single heat spot cooling of 5 G chip is fabricated utilizing this electrocaloric composite and electromagnetic actuation. The continuous three-dimensional (3-D) thermal conductive network embedded in the polymer acts as nucleation sites of the ordered dipoles under applied electric field, efficiently collects thermal energy at the hot-spots arising from field-driven dipolar entropy change, and opens up the high-speed conduction path of phonons. The synergy of two components, thus, tackles the challenge of sluggish heat dissipation of the electroactive polymers and their contact interfaces with low thermal conductivity, and more importantly, significantly reduces the electric energy for switching the dipolar states during the electrocaloric cycles, and increases the manipulable entropy at the low fields. Such a feasible solution is inevitable to the precisely fixed-point thermal management of next-generation smart microelectronic devices.

## Abstract (web summary)

Efficient thermal structure and precise heat management become a substantial challenge for electronics. Here, authors utilize the synergistic effect of classic heat transfer and electrocaloric cooling for fixed-point thermal management of chips.

## Introduction

The pressure to decrease the size and increase the switching frequency of modern microelectronics, such as fifth-generation (5 G) chips, lead to tremendous power dissipation density that limits the device performance, and have made thirsty for the high-efficient thermal manage systems^1–3^. Most conventional cooling technologies of high-power modules utilize heat transfer to external heat sink or cold plate by forced circulation of air or liquid^4^. Most recently, a directed heat pumping system of liquid microfluidics realizes large heat transfer at low energy consumption and green environmental protection^5^. Due to the multilayer architectures in the heat flow path and inevitable thermal resistance at interface, such passive cooling systems are hard to achieve rapid heat transfer, even with the aid of thermal interface materials. What is even worse is that they face the challenge of efficient cooling with low-temperature differentials, thus require extra cooling units^6^.

Electrocaloric cooling is an active and solid-state refrigeration technology with zero-global warming potential, high efficiency, environmentally benign, and easy miniaturization^7–13^. It exploits the reversible thermal change of ferroelectric materials with the application or removal of an electric field. Most recently, polymeric ferroelectrics have been extensively investigated because of the excellent electrocaloric performance, flexibility, and facilitation in the large-scale fabrication^14–16^. Nevertheless, little attention has been paid to their inherent low thermal conductivity, which is arising from the complicated chain conformation in the crystalline regions, and random arrangement or entanglement of the chains in the amorphous domains. During the high-speed switching between the heat sink and source, such electrocaloric active layer with low thermal conductivity results in insufficient depth of heat penetration of the refrigeration device^17,18^. That is why the cooling performance decreases rather than increases when the operation frequency is increased in the early reports^19^.

The construction of a continuous three-dimensional thermal conductivity network in a polymer matrix has proven to be an effective and simple way to improve the thermal transport properties of materials for passive thermal management systems^20^. Here, we demonstrate a general and practical method on thermal management of 5 G chip by a device prototype with synergistic effects of passive heat-conductive network and active electrocaloric refrigeration. We combine the electrocaloric polymer with a 3-D network of ferroelectric ceramic to afford an interpenetrating and heterogeneous composite. When an electric field is applied to the composite, it induces a disorder-to-order orientational change of the electric dipoles in the polymer. Interaction of the oriented dipoles leads to nanoscale regions and release the heat to surround area^21^. These “hot spots” are randomly distributed in the thermal-insulated polymer. The successive heat-conductive ceramic network embedded in the polymer matrix provides large interfacial contact area and constructs a high-speed path for the phonon transportation; thus, it can achieve rapid and directional delivery of thermal energy at the moment of the applied electric field. Relaxor-type ferroelectric polymer exhibits a large electrocaloric effect in the operation temperature range (20-60 °C) of semiconductor chips. Its electrocaloric effect can further be enhanced by introducing ferroelectric ceramic network. The electroactive composite material exhibits a 240% increase in electrocaloric performance and a 300% enhances in thermal conductivity, compared to the neat polymer. We also develop a device prototype of the scaled-up version for a single heat spot cooling of 5 G chip utilizing this electrocaloric material and electromagnetic actuation. The maximum heat fluxes for heating and cooling are achieved at an electric field as low as 30 V μm^−1^, which ensures the active cooling device to be operated properly at the maximum supply voltage of semiconductor chips. The feasible solution is valuable to the precisely fixed-point thermal management of the next-generation smart microelectronic devices.

## Results

### Large electrocaloric performance of 3-3 PCC

**Fig. 1:**
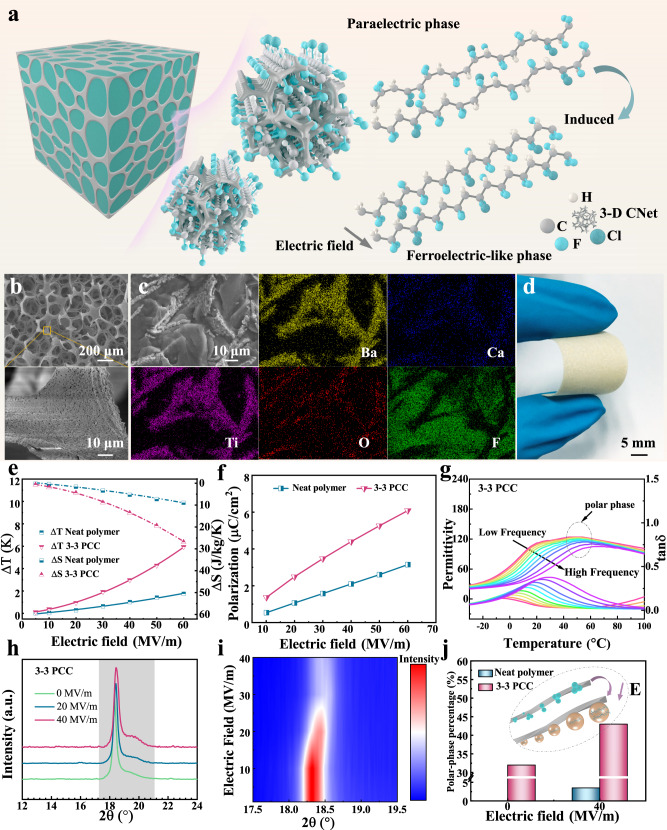
Structural origins of enhancing electrocaloric performance by 3-D thermally conductive networks. **a** Schematic diagram of inducing the transition from paraelectric (nonpolar) to ferroelectric-like (polar) phases for the polymer in a restricted state-space. **b** SEM images of 3-D CNet. **c** The element mapping of Ba, Ca, Ti, O, and F, confirmed that the continuous 3-D CNet is implemented in 3-3 PCC. **d** Optical image of 3-3 PCC. **e** The ΔS and ΔT of the neat polymer and 3-3 PCC as a function of applied electric field. **f** Polarization versus electric field for the neat polymer and 3-3 PCC. **g** The temperature dependence of the dielectric permittivity and loss (*tanδ*) of the 3-3 PCC. **h** In situ XRD patterns of the 3-3 PCC with increasing electric field. **i** In situ XRD intensity mapping of the 3-3 PCC as a function of 2Θ and electric field. **j** The histogram of polar phase ratio at 0 and 40 MV m^−1^. The inset of the elliptical dashed area shows a schematic diagram of the 3-D CNet acting as a polar nucleation site and gradually growing and expanding with this site as the core under an external electric field.

Electrocaloric effect (ECE), the thermal effect of a dielectric material caused by the manipulation of an electric dipole by an applied electric field under adiabatic conditions^22,23^. The introduction of 3-D ceramic network (3-D CNet) into the polymer matrix leads to a restricted state of molecular chains in the interfacial regions and gaps of the network, which results in the conversion of non-polar conformation molecular chains to polar conformation^24–26^ (Fig. 1a). This increases the entropy of the dipole that can be driven by a low electric field. For the first time, we have cleverly combined traditional passive heat transfer with active electrocaloric cooling in 3-3 ferroelectric polymer/ceramic composite (3-3 PCC) materials. The fabrication and structure of 3-3 PCC materials are discussed in Supplementary Note 2. To test our concept, we chose lead-free ferroelectric ceramic Ba_0.85_Ca_0.15_Zr_0.1_Ti_0.9_O_**3**_ (BCZT) as a continuous 3-D CNet (Fig. 1b) in the poly(vinylidene fluoridetrifluoroethylene-chlorofluoroethylene) (P(VDF-TrFE-CFE)) polymer matrix, which has been reported to have excellent dielectric, ferroelectric, and piezoelectric properties^27^. Cross-sectional SEM element mapping of the 3-3 PCC demonstrates that continuous 3-D ceramic thermal conductivity pathways are successfully constructed in the 3-3 PCC materials (Fig. 1c). In addition, the 3-3 PCC has good flexibility (Fig. 1d), which facilitates its good contact with the chip surface for heat to transfer out.

In order to substantiate the excellent performance of 3-3 PCC materials, we characterize the ECE of the sample using an in situ calibrated measurement system (Supplementary Note 3). The isothermal entropy change (Δ*S*) and adiabatic temperature change (Δ*T*) can be derived from the following equations:^28–30^

1 $${{{{{\rm{Q}}}}}}=T\varDelta S={\int }_{T}^{T+\varDelta T}{C}_{{{{{{\rm{p}}}}}}}\,dT$$

Where *T* is environmental temperature*. C*_P_ is the specific heat capacity. Apparently, the electrocaloric properties are significantly enhanced under a low electric field after introducing the 3-D CNet into the polymer. For instance, an isothermal cooling energy density *Q* of up to 14.72 MJ m^−3^ with an electrocaloric strength *Q*/Δ*E* of 245.3 kJ m^−2^ MV^−1^ is achieved at 60 MV m^−1^. It is comparable to the results reported recently in blend and composite materials^14,24^. Simultaneously, the 3-3 PCC at 60 MV m^−1^ displays a ΔS of 26.76 J kg^−1^ K^−1^, a Δ*T* of 5.94 K, a ΔS/ΔE of 446 J mm kg^−1^ K^−1^ MV^−1^, and a ΔT/ΔE of 99 K mm MV^−1^ (Supplementary Fig. 6b–f**)**, which is 240% higher than the state-of-the-art electrocaloric polymers at the same field strength (Fig. 1e). As mentioned above, the system entropy of 3-3 PCC is significantly increased after introducing the 3-D thermal conductivity network in the polymer. This leads to a manipulable entropy augmentation under low electric field, which generates a large ECE. According to the thermodynamic Landau–Devonshire theory, Δ*S* = *−*$\frac{1}{2}\beta P^2$, where *β* is a coefficient and *P* is the polarization strength^31^. The Δ*S* is proportional to the quadratic of *P*. Compared with the neat polymer, the value of maximum polarization (*P*_*max*_) for 3-3 PCC reaches 6.08 μC cm^−2^ at 60 MV m^−1^, which is about twice that of the polymer under the same field strength (Fig. 1f and Supplementary Fig. 7). Therefore, in this system, the significant enhancement of ECE is related to the modulation of polarization performance after inserting 3-D CNet into the polymer.

The temperature dependent dielectric properties of the 3-3 PCC are presented in Fig. 1g. A weak frequency-independent peak is observed at 50 °C compared to the neat polymer (Supplementary Note 4). Earlier studies show its correlation with the polar phase. ^28^ This suggests that the introduction of 3-D CNet into the polymer can induce the formation of polar nano-regions. We further demonstrate that the presence of polar nanodomains in 3-3 PCC at zero field strength can significantly reduce the potential barrier for nucleation growth of polar phases (Supplementary Note 5). The changes in the polar phase of the neat polymer and 3-3 PCC with increasing electric field are investigated using in situ XRD. In the neat polymer, only a slight decrease in polar phase is detected with increasing electric field; and almost no increase in polar phase diffraction peaks is noticed (Supplementary Fig. 11a). In contrast, in the 3-3 PCC, as the electric field increases, the diffraction peaks of the non-polar phase progressively decrease, while the diffraction peaks of the polar phase gradually increase (Fig. 1h, i). At the same time, both diffraction peaks are shifted to high angles with the increase of electric field. Quantitatively, the 3-3 PCC shows that volume fraction of the polar phase increases from the initial 32% to 43% when the electric field is increased to 40 MV m^−1^; while for the neat polymer, the volume fraction is only 3.5% (Fig. 1j and Supplementary Fig. 12).

Thus, the enhanced ECE of the composite at low fields can be ascribed to the increased polar phases of the polymer in the presence of 3-D CNet. The introduction of 3-D CNet into the polymer matrix increases the number of polar nanodomains. It also enhances interfacial areas of the polar/nonpolar phases and ceramic-network/polymer, resulting in increased polarization performance (manipulable entropy) at low fields. The energy required for nucleation of polar domains is usually higher than that for domain growth. Therefore, in the presence of polar nanodomain as nucleation center, a small electric field is enough to make the polar domains expand and grow outwards, which causes the large ECE under low field. In other words, 3-D CNet acts as a polar nucleation site. Simultaneously, 3-D CNet also serves as a continuous heat transfer path. When an electric field is applied and withdrawn, the polar domains expand around the polar nano-nucleus located on the 3-D CNet (Fig. 1j inset of the dotted ellipse area). This allows the generated heat/cold to be quickly transferred to the heat sink/heat source using the 3-D CNet thermal conductivity network as a bridge.

### Modulation of thermal conductivity

**Fig. 2:**
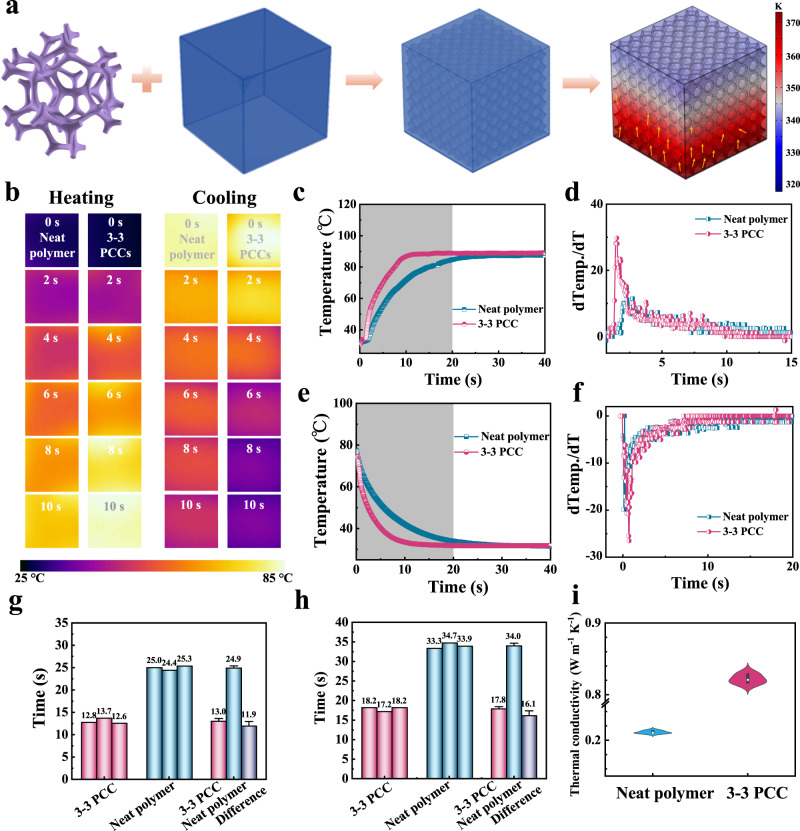
Excellent thermal conductivity of 3-3 PCC materials. **a** Schematic diagram of the optimized 3-D heat conduction structure by finite element simulation. **b** Infrared thermal images at selected time. **c**, **e** The curve of temperature versus time and **d**, **f** the partial differential of temperature versus time during heating and cooling. **g**, **h** Statistical histogram of the average temperature rise and fall time of three times when reaching a stable value. **i** Thermal conductivity.

The effect of introducing the 3-D thermal conductivity network on the passive heat transfer behavior of the polymer is investigated by finite element method. Firstly, we simulate the heat transfer behavior of the thermally conductive fillers discontinuously dispersed in the polymer or in a form of 3-D networks. The 3-D network structure provides a continuous phonon pathway and substantially improves the passive heat transfer performance of the 3-3 PCC (Fig. 2a, Supplementary Note 6). We put the neat polymer and 3-3 PCC with the same sizes on the 85 °C hot stage to reach a constant surface temperature, and then transfer them to the cold plate. The temperature change of the sample surface is recorded by thermal infrared imager in real time. Compared to the neat polymer, the 3-3 PCC has superior performance of passive heat transfer (Fig. 2b). The dynamic evolution process shows their differences more clearly (Supplementary Movie 1). The temperature versus time profiles during heating and cooling processes are shown in Fig. 2c, e. We can clearly see that the 3-3 PCC reaches the temperature maximum or drops to room temperature in a shorter time that the neat polymer.

The first-order partial derivative of temperature to time is related to the heating and cooling rates. They reach the maximum value (negative lowest point) at the initial heating (cooling), and then gradually decrease (the negative value gradually tends to zero, Fig. 2f) over time (Fig. 2d). Regardless of whether it is heating or cooling, the utmost first-order partial derivative is observed in the 3-3 PCC samples. This further verified its excellent thermal conduction. The average time of 3-3 PCC heating experiment is 11.9 s faster than that of neat polymer, and the cooling rate is 16.1 s faster (Fig. 2g, h). Then, we detect the thermal conductivity in the thickness direction of the corresponding films by flash DSC (Fig. 2i). The thermal conductivity of the neat polymer films is 0.21 W m^−1^ K^−1^. The 3-3 PCC exhibits thermal conductivity of 0.84 W m^−1^ K^−1^, indicating a 300% enhancement over the neat polymers. The strategy of using a three-dimensional network to enhance passive heat transfer can yield a high thermal-conductive material suitable for electrocaloric refrigeration. This makes it possible to enhance both passive heat dissipation and active cooling material in the same system.

### Electrocaloric refrigeration device prototypes for chip cooling

**Fig. 3:**
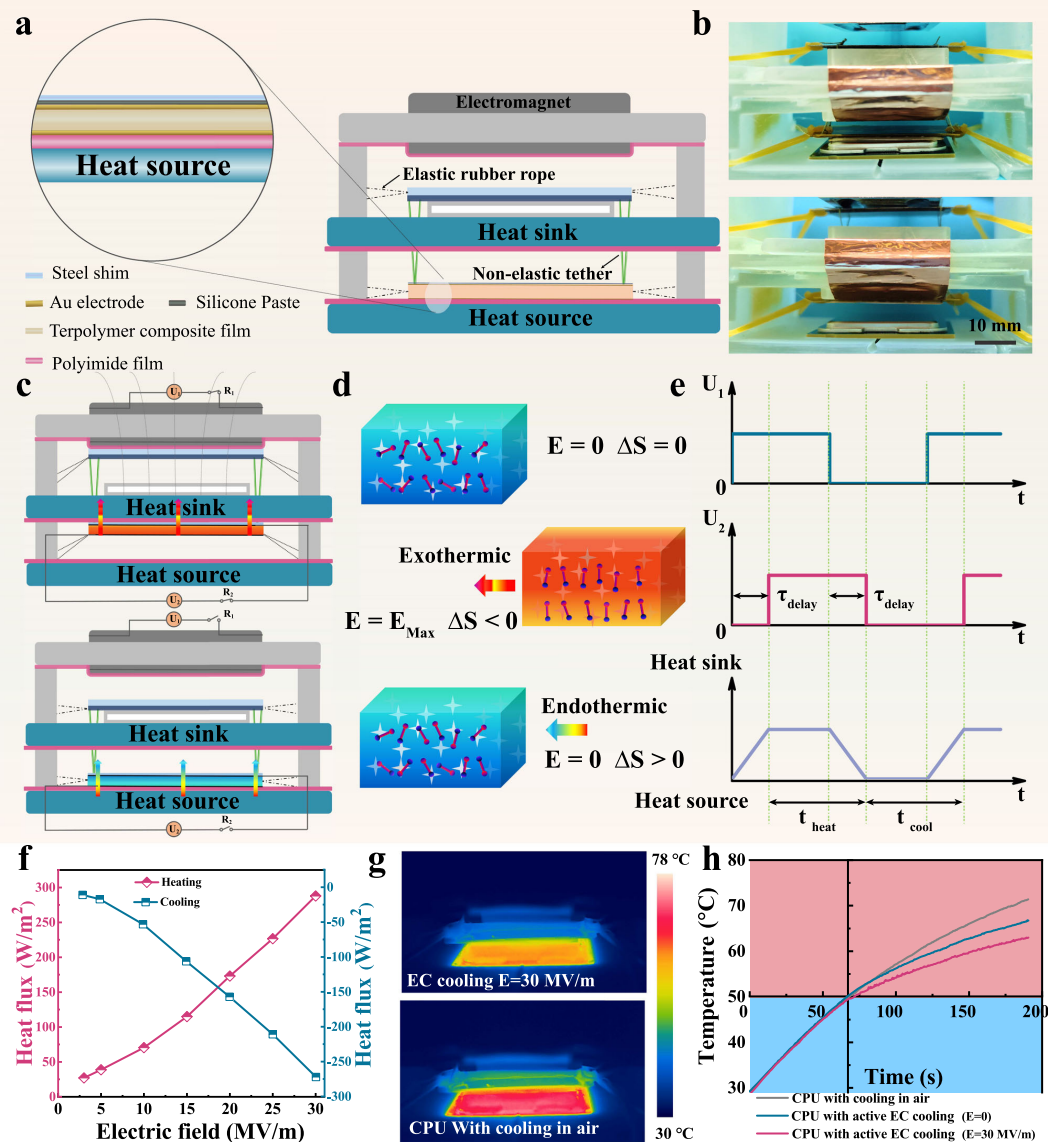
A solid-state electrocaloric cooling device. **a** Schematic illustration of the electrocaloric polymer stack and solid-state cooling device. **b** Photograph of the active EC device, the elaborated main framework was obtained by 3-D printing. **c** The schematic shows how an electromagnetic field drives an electrocaloric polymer stack to move heat from a heat source to a heat sink. The high-speed heat transfers from heat source to sink can be achieved by associating the active cooling of electrocaloric polymer stack with heat dissipation cycle. **d** The working mechanism of the ECE based on the change of dipole entropy. **e** Time domain illustration of the cooling cycle. **f** The maximum heat flux of the electrocaloric stack on the heating and cooling side versus the applied electric field is measured by a heat flux sensor at an operation frequency of 0.1 Hz. **g** Infrared thermal images of CPU in active electrocaloric cooling. **h** Temperature versus time curves of CPU in air, by active electrocaloric cooling (U_1_ = 12 V at 1 Hz, U_2_/d_2_ = *E* = 0 MV m^−1^, d_2_ is the thickness of the electrocaloric cooling stack) and active electrocaloric cooling (U_1_ = 12 V at 1 Hz, *E* = 30 MV m^−1^).

Then we demonstrate the advantage of the large ECE of 3-3 PCC materials at low electric fields. An electrocaloric cooler exploiting electromagnetic drive is designed for active chip cooling. In order to avoid mutual interference between the drive and active cooling modules, these two are effectively separated by an external 3-D printed frame. The active cooler mainly consists of electromagnet, magnetizable steel shim, heat sink, electrocaloric stack, and heat source from top to bottom (Fig. 3a). Photographs of the electrocaloric refrigeration device that switches periodically between heat sink (top) and heat source (bottom) is shown in Fig. 3b. First, switching on the electric relay R_1_, the electromagnet generates a magnetic field that attracts the steel piece at the top to move upward, while under the traction of the non-elastic tether, the electrocaloric stack at the bottom will move upward in parallel to make contact with the heat sink. After that, the relay R_2_ is turned on. A given electric field E is applied to both sides of the electrocaloric stack. The dipoles in the electrocaloric stack are ordered in the direction of the electric field, so that the entropy inside the material decreases sharply with an increase in temperature. The heat from electrocaloric layer is transferred upward to the heat sink through the upper metal. After the heat transfer is completed, the relay R_1_ is switched off and the electromagnetic field disappears. The electrocaloric stack springs back to the lower heat source under gravity and elastic force. Subsequently, relay R_2_ is disconnected, and the electric field applied on electrocaloric stack is removed. The dipole in the active layer returns to the disordered state and the material entropy increases. When the temperature decreases, the electrocaloric stack absorbs heat from the lower heat source to achieve cooling (Fig. 3c, d). It is worth noting that during the whole process, the electrocaloric cooler pumps heat from the bottom heat source to the top heat sink, completing a single cycle of active cooling. To make sufficient contact between the electrocaloric stack and the cold/heat source before applying/removing the electric field to the electrocaloric cooling layer, the switching time of the relay R_2_ is always 0.1 s later than that of the relay R_1_ (Fig. 3e). Furthermore, electrocaloric cooling devices driven by electromagnetic fields can be operated at different frequencies (Supplementary Movie 2 and 3).

For practical application, the active electrocaloric cooling device should be operated properly at the chip’s maximum supply voltage of 12 V. Considering 0.4 μm thick multilayer film capacitors are easily achieved by a biaxial stretching process, the device needs to accomplish active chip cooling at a low field of 30 MV m^−1^. Related work has not been reported in studies so far. The maximum heat flux of the electrocaloric stack on the heating and cooling side versus the applied electric field is measured by a heat flux sensor at an operation frequency of 0.1 Hz (Fig. 3f). The maximum heat fluxes for heating and cooling at the electric field of 30 MV m^−1^ are 288 and −272 W m^−2^, respectively. Supplementary Fig. 16a shows the effect of operating frequency on the average heat flux. An average cooling heat flow of −213 W m^−2^ can be achieved at a frequency of 1 Hz. Furthermore, the average cooling heat flux can be further increased at higher frequencies (−236 W m^−2^ at a frequency of 1.25 Hz). Similarly, the ideal temperature span (1.1 K) of the electrocaloric cooling device is obtained at a frequency of 1 Hz with an electric field of 30 MV m^−1^ (Supplementary Fig. 16b). Finally, we use a ceramic heating plate to warm it to simulate the overheating state of the real central processing unit (CPU) chip in the working process. The active electrocaloric cooler is placed on top of the heating chip to evaluate its cooling performance (Supplementary Note 7). CPU surface temperature is measured using an infrared thermal imager (Fig. 3g). The start-up temperature of the electrocaloric device is 50 °C, considering that higher temperature will reduce the efficiency of the CPU in practice. After starting the electrocaloric cooler (U_1_ = 12 V at 1 Hz, *E* = 30 MV m^−1^), the surface temperature of the chip, which is originally cooled in the air, dropped from 71.4 °C to 63 °C (Fig. 3h, Supplementary Note 8). The stability of the chip performance is related to the operating temperature. When the chip operating temperature is close to 70–80 °C, the risk of chip failure increases by 10% for every 2 °C increase in temperature. The above results show that the electrocaloric cooler can maintain the chip temperature (63 °C) away from the range of the high risk of failure (70 < 71.1 < 80 °C). It further demonstrates that active electrocaloric cooling can achieve cooling of the 5 G chip in a precise, efficient, and scalable manner.

## Discussion

In summary, we demonstrate a general and practical method to modulate the thermal conductivity and electric refrigeration performance of the relaxor-type ferroelectric polymer by using 3-D lead-free ferroelectric ceramic interpenetrating networks. The introduction of 3-D CNet into the polymer matrix increases the number of polar nanodomains. It also enhances interfacial areas of the polar/nonpolar phases and ceramic-network/polymer, resulting in increased manipulable entropy at low fields. On the other hand, the continuous 3-D network structure opens up the high-speed thermal conduction path of phonons at the “hot spots” formed by the nucleation of nanodomains, which enables the rapid cold/heat transport in the electrocaloric layer. The resulting material exhibits a 240% increase in electrocaloric performance and a 300% enhances in thermal conductivity compared to the neat polymer. We develop a scaled-up version of the device prototype for a single heat spot cooling of 5 G chip utilizing this electrocaloric composite and electromagnetic actuation mechanism. A feasible solution is provided for the precisely fixed-point thermal management of next-generation smart microelectronic devices.

## Methods

### Preparation of P(VDF-TrFE-CFE) films

P(VDF-TrFE-CFE) (62.1/30.1/7.8 mol%, Piezotech, France) was dissolved in N, N-dimethylformamide (DMF, 99.8%, Aladdin) and stirred for 12 h to obtain a homogeneous solution at a concentration of 4 wt.%. Then, the solutions were cast onto a glass substrate and placed in an oven at 60 °C for 24 h to remove the solvent. Subsequently, the obtained films were annealed in a vacuum oven at 106 °C for 10 h to improve the crystallinity. The resulting film thickness was in the range of 15 µm to 20 µm.

### Fabrication of three-dimensional ceramic networks

First, 10.5 ml ethanol, 4.59 ml acetylacetone and 27 ml glacial acetic acid were mixed to obtain a co-solvent. After that, tetrabutyl titanate (7.14 g, 99%, Aladdin), barium hydroxide (3.71 g, 99.99%, Aladdin), calcium hydroxide (0.18 g, 95%, HUISHI), and zirconium acetylacetonate (1.60 g, 99 wt. %, Aladdin) were sequentially dissolved in the co-solvent and stirred to form a uniform solution to obtain a BCZT sol. Next, the polyurethane foam template (TX704, ITW Texwipe) was immersed in the BCZT sol for 10 min. Then, the excess sol was squeezed out from the polyurethane frame and dried at 55 °C to obtain the BCZT precursor template. Finally, the 3-D CNet was obtained by the BCZT precursor template after calcine at 1200 °C for 2 h.

### Preparation of 3-3 polymer/ceramic composites

P(VDF-TrFE-CFE) (62.1/30.1/7.8 mol%, Piezotech, France) was dissolved in DMF and stirred for 12 h to obtain a homogeneous solution at a concentration of 15 wt.%. After that, the 3-D CNet was placed on a PTFE mold with a length, width, and height of 40*40*20 mm. The solution of P(VDF-TrFE-CFE) in DMF (15 wt.%) was filled into the 3-D CNet and placed in an oven at 60 °C for 0.5 h. Then, repeat the above procedures several times until the 3-D CNet was completely filled by the polymer matrix. The resulting composite was dried at 60 °C for 12 h to remove excess solvent. Next, 3-3 PCC materials were obtained by converting the aforementioned materials to a dense body through hot-pressing at 140 °C for 10 min, under a pressure of 0.7 MPa. A 100 µm thick P(VDF-TrFE-CFE) film was placed on the top and bottom surfaces of the hot-pressed precursor as a buffer layer. The typical thickness after hot-pressing was around 800 µm. Subsequently, the samples are thinned by abrasive paper to achieve the desired thickness. Finally, the resulting 3-3 PCC films were annealed in a vacuum oven at 106 °C for 10 h to improve the crystallinity of the films.

### Fabrication of the electrocaloric stack

A 35*25 mm mask plate was placed on both sides of the prepared 3-3 PCC film. The two mask plates were misaligned by 5 mm along the long side to reserve the electrode connection area. Bilateral gold electrodes were deposited by a high vacuum coating system (Leica EM ACE600). Then silver wires were used to lead out the electrodes for high-voltage power connection. Two-component conductive silver paste was used as a binder (8821X, SHENGGELU TECHNOLOGY). Finally, the electrocaloric stack was encapsulated with polyimide tape to achieve electrical insulation.

### Fabrication of the electrocaloric cooling device

The volume of the active area of a typical electrocaloric stack is 30*25*0.1 mm. The edges were attached to a 40*20*0.12 mm magnetic steel piece by double-sided adhesive tape. The middle active area was filled with high thermal conductivity silicone (OMEGATHERM 201). The body frame of the cooler was obtained by commercial 3-D printing equipment. It mainly consists of two modules, the drive and active cooling modules, and comes with electromagnet installation position, chip cooling zone, and wiring hole. The magnetizable steel shim (40*20*0.35 mm) and the electrocaloric active layer were attached to the bottom of the corresponding module of the resin frame by latex wires respectively. The two steel pieces in the drive and cooling modules were connected to each other by tethers. A piece of Mn-Zn ferrite soft magnetic material was glued to the lower side of the steel shim used in the driver module to increase the magnetism. Finally, the commercial electromagnet was installed in the upper part of the device to complete the assembly of the electric cooler.

### Characterizations

The microstructure was observed using scanning electron microscope (SEM, SSX-550 Shimadzu, Japan). X-ray energy spectrum analyzer (EDX) was performed to identify the 3-D CNet in the polymer matrix. The crystal structure was analyzed by X-ray diffractometer (XRD, Bruker D8 ADVANCE). The crystallinity and crystal size were evaluated by differential scanning calorimetry (DSC, X3 DSC, TA Instruments). The evolution of the conformational structure after introducing the 3-D thermal conductivity network into the polymer was detected by Fourier Transform Infrared Spectroscope (FTIR, Nicolet iS10, Thermo Fisher Scientific). The temperature change of the sample and chip surface was recorded in real time by an infrared thermal imager (226 s, FOTRIC). The dielectric properties were measured by broadband dielectric and impedance analyzer (Concept80, Novocontrol Technologies, Germany). Ferroelectric hysteresis loops were characterized by Ferroelectric Test Systems (Premier II, Radiant Technologies, USA). Thermal conductivity of the sample was characterized using fast scanning chip-calorimeter (Flash DSC 1, Mettler-Toledo, Switzerland). The ECE heat calibration and the electrocaloric stack cooling and heating heat flow are captured by the heat flux sensor (HFS-4, OMEGA). The high voltage was generated by a high voltage power module (LNC 30000, Heinzinger). The low voltage system was produced by high-precision digital source meter (B2902A, KEYSIGHT).

### Reporting summary

Further information on research design is available in the Nature Research Reporting Summary linked to this article.

## Supplementary information

Supplementary Information

Peer Review File

Description of Additional Supplementary Files

Supplementary Movie 1

Supplementary Movie 2

Supplementary Movie 3

Reporting Summary

## Data Availability

The data that support the findings of this study are available from the corresponding authors upon reasonable request.
