# Supplementary material for: Thermal management of chips by a device prototype using synergistic effects of 3-D heat-conductive network and electrocaloric refrigeration
Source: Nat Commun. 2022 Oct 4;13:5849. doi: 10.1038/s41467-022-33596-z (PMC9532434; doi:10.1038/s41467-022-33596-z)
Supplement: Supplementary file 1 — Supplementary Information [file 41467_2022_33596_MOESM1_ESM.pdf]

Supplementary information

Thermal Management of Chips by a Device Prototype Using Synergistic Effects of 3-D Heat-Conductive Network and Electrocaloric Refrigeration

Ming-Ding Li[1], Xiao-Quan Shen[1], Xin Chen[2], Jia-Ming Gan[1], Fang Wang[1], Jian Li[3], Xiao-Liang Wang[1], Qun-Dong Shen[1*]

1. Department of Polymer Science and Engineering, MOE Key Laboratory of High Performance Polymer Materials and Technology, School of Chemistry and Chemical Engineering, Nanjing University, Nanjing 210023, China.
2. Materials Research Institute, The Pennsylvania State University, University Park, Pennsylvania 16802, USA.
3. State Key Laboratory of Analytical Chemistry for Life Science, School of Chemistry and Chemical Engineering, Nanjing University, Nanjing 210023, China

*Corresponding authors: qdshen@nju.edu.cn

Table of contents

Supplementary Note 1. Supplementary methods and characterizations

Supplementary Note 2. Manufacturing and structure of 3-3 PCC materials

Supplementary Note 3. Electrocaloric effect measurement with *in situ* calibration

Supplementary Note 4. Relaxor ferroelectric behavior and dielectric properties of 3-3 PCC materials

Supplementary Note 5. Structural origins of large electrocaloric performance

Supplementary Note 6. Heat transfer process simulated by finite element methods

Supplementary Note 7. An active electrocaloric cooling devices for chip thermal management

Supplementary Note 8. Infrared thermal image of CPU under different cooling conditions

Supplementary References

Supplementary Note 1. Supplementary methods and characterizations.

Preparation of Photo-induced force microscopy samples

First, 13 ml glacial acetic acid, 1 ml acetylacetone, and 1 ml 2-ethoxyethanol were mixed to obtain a co-solvent. After that, tetrabutyl titanate (0.928 g, 99%, Aladdin), zirconium butoxide solution (0.144 g, 99 wt. %, Aladdin), barium acetate (0.658 g, 99%, Aladdin), and calcium acetate (0.072 g, 99%, J&K Scientific) were sequentially dissolved in the co-solvent and stirred to form a uniform solution to obtain a 0.2 M $Ba_{0.85}Ca_{0.15}Zr_{0.1}Ti_{0.9}O_3$ (BCZT) sol. After 24 h of static aging, the sol was spin-coated onto the SiO_2/Si substrate cleaned by ethanol and acetone at a rate of 4000 r min^{-1}. Following each spin-coating, the wet film was dried at 200 °C for 10 min and then pyrolyzed on a hot plate at 400 °C for 5 min. The spin-coating process was repeated five times and finally annealed in an alumina crucible at 700 °C for 30 min to obtain the BCZT/SiO_2/Si film. Afterwards, P(VDF-TrFE-CFE) (62.1/30.1/7.8 mol%, Piezotech, France) was dissolved into 2-butanone to make a solution of 10 mg ml^{-1}. The obtained solution was spin-coated onto the BCZT/SiO_2/Si films at 3000 r min^{-1} for 40 Subsequently, the resulting films were annealed in a vacuum furnace at 106 °C for 10 h to obtain P(VDF-TrFE-CFE)/BCZT/SiO_2/Si (sample B) films. A 2-butanone solution of P(VDF-TrFE-CFE) at 10 mg ml^{-1} was spin-coated directly onto the SiO_2/Si substrate cleaned by ethanol and acetone, and then annealed in a vacuum furnace at 106 °C for 10 h to obtain P(VDF-TrFE-CFE)/SiO_2/Si (sample A) films.

Characterization.

Atomic force microscope (AFM) and photo-induced force microscopy (PiFM) were recorded on VistaScope microscope from Molecular Vista Inc.. The attachments were coupled using a QCL laser system with a tuning range of 770 to 1885 cm^{-1} and a wave number resolution of 1 cm^{-1}. The microscope was operated in dynamic mode, with NCH-Au 300 kHz noncontact cantilevers from Nanosensors. To obtain the temperature span, the copper foil (100 μm) was used as heat sink. All heat loads at the bottom were removed to reduce heat loss. Only polyimide membrane (60 μm) was used as the

bottom heat source layer for infrared imaging. The electrocaloric cooler pumps heat from the bottom heat source (polyimide membrane) to the top heat sink (copper foil). Now, time versus temperature curve for polyimide recorded by infrared imaging is the cooling curve. Then, under normal operating conditions, the electric relay R_1 in the control circuit was delayed for half a cycle to turn on, and R_2 remains unchanged (**Fig. 3e**). At this time, the heat was pumped from the copper foil to the polyimide film. The recorded time versus temperature curve responds to the ideal heating curve for the temperature span.

Supplementary Note 2. Manufacturing and structure of 3-3 PCC materials.

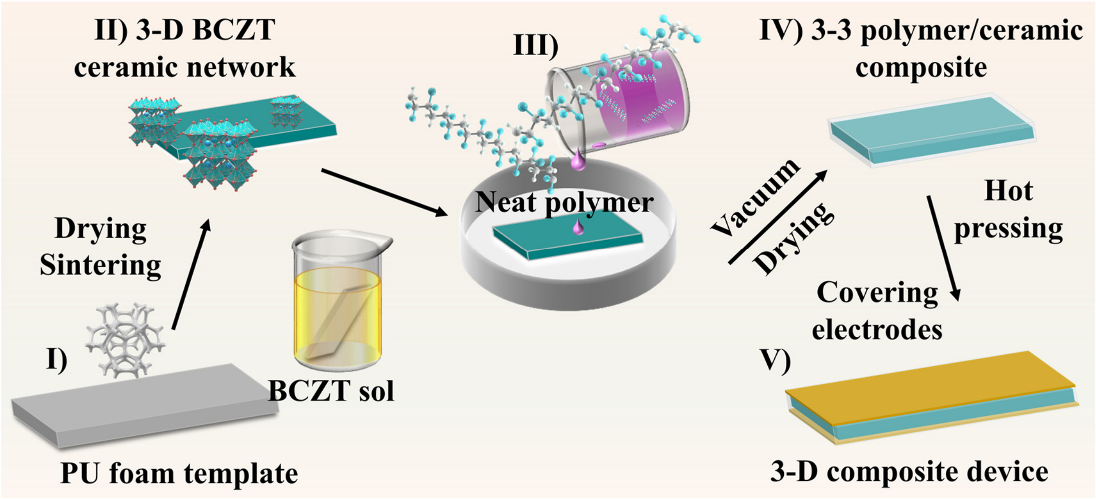

Supplementary Fig. 1. Preparation of 3-3 polymer/ceramic composite (3-3 PCC) materials. Schematic diagram of the 3-3 PCC manufacturing.

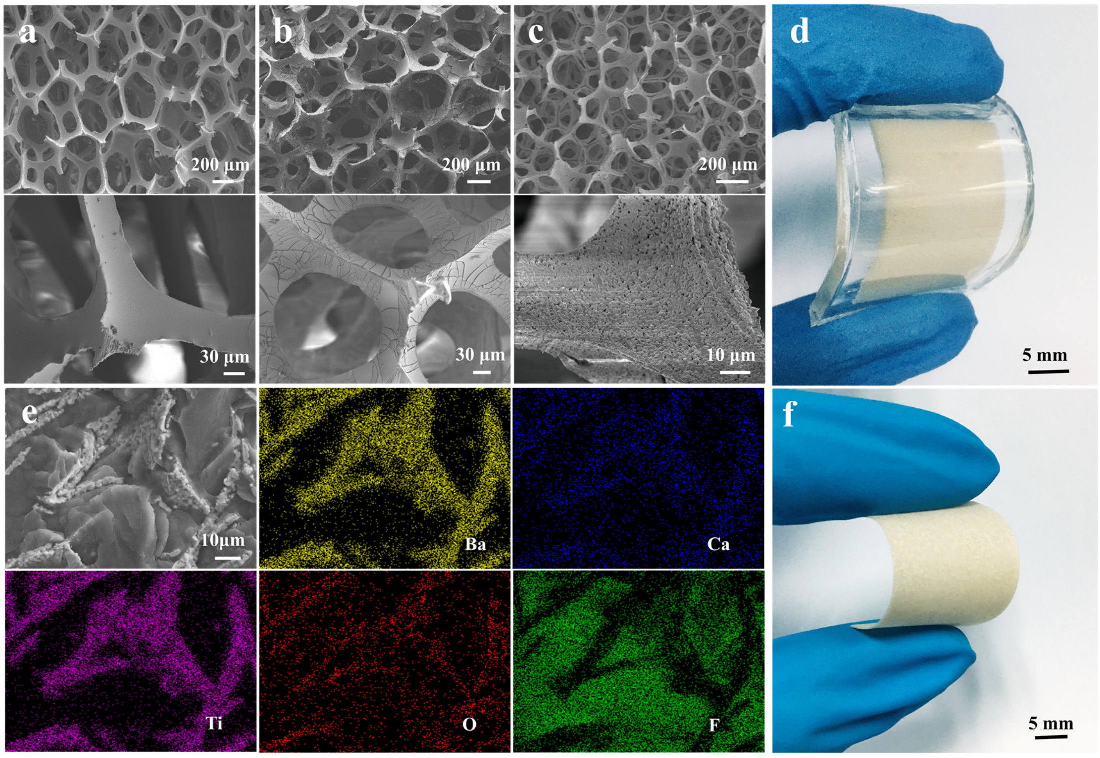

Supplementary Fig. 2. Manufacturing and structure of 3-3 PCCs. a) Scanning electron microscope (SEM) images of polyurethane (PU) foam. **b**) SEM images of PU foam after dipping BCZT sol and drying. **c**) SEM images of three-dimensional ceramic network (3-D CNet). **d**) Optical image of the 3-D CNet encapsulated by polydimethylsiloxane (PDMS). **e**) The element mapping of Ba, Ca, Ti, O and F, confirmed that the continuous 3-D CNet is implemented in the 3-3 PCC. **f**) Optical image of the 3-3 PCC.

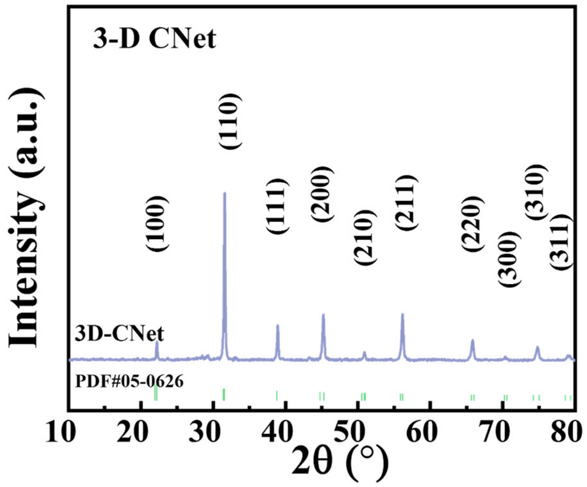

Supplementary Fig. 3. Crystal structure of the 3-D CNet. X-ray diffraction pattern of the 3-D CNet.

Supplementary Fig. 1 shows the schematic diagram of the 3-3 PCC manufacturing. The continuous 3-D CNet is obtained by calcining the PU foam template after dipping into the precursor solution (**Supplementary Fig. 2a-c**). The interspersed and overlapped ceramic grain structures are observed in the local magnification patterns, and are beneficial for the rapid thermal transportation. Furthermore, X-ray diffraction result shows that the 3-D CNet has a typical perovskite structure without any heterogeneous phases (**Supplementary Fig. 3**). Optical image of a 3-D CNet encapsulated by polydimethylsiloxane (PDMS) further demonstrates the integrity and flexibility, indicating that we have successfully prepared a self-supporting 3-D CNet (**Supplementary Fig. 2d**). Cross-sectional SEM element mapping of the 3-3 PCC demonstrates that continuous 3-D ceramic thermal conductivity pathways are successfully constructed in the 3-3 PCC materials (**Supplementary Fig. 2e**). The SEM mapping of the corresponding Ba, Ca, Ti, O, and F elements further clearly verifies the presence of a continuous 3-D ceramic structure. The interconnection network structure of the ceramic network in the 3-3 PCC indicates the possibility of achieving high thermal conductivity. In addition, the 3-3 PCC has good flexibility (**Supplementary Fig. 2f**), which facilitates its good contact with the chip surface for heat to transfer out.

Supplementary Note 3. Electrocaloric effect measurement with *in situ* calibration

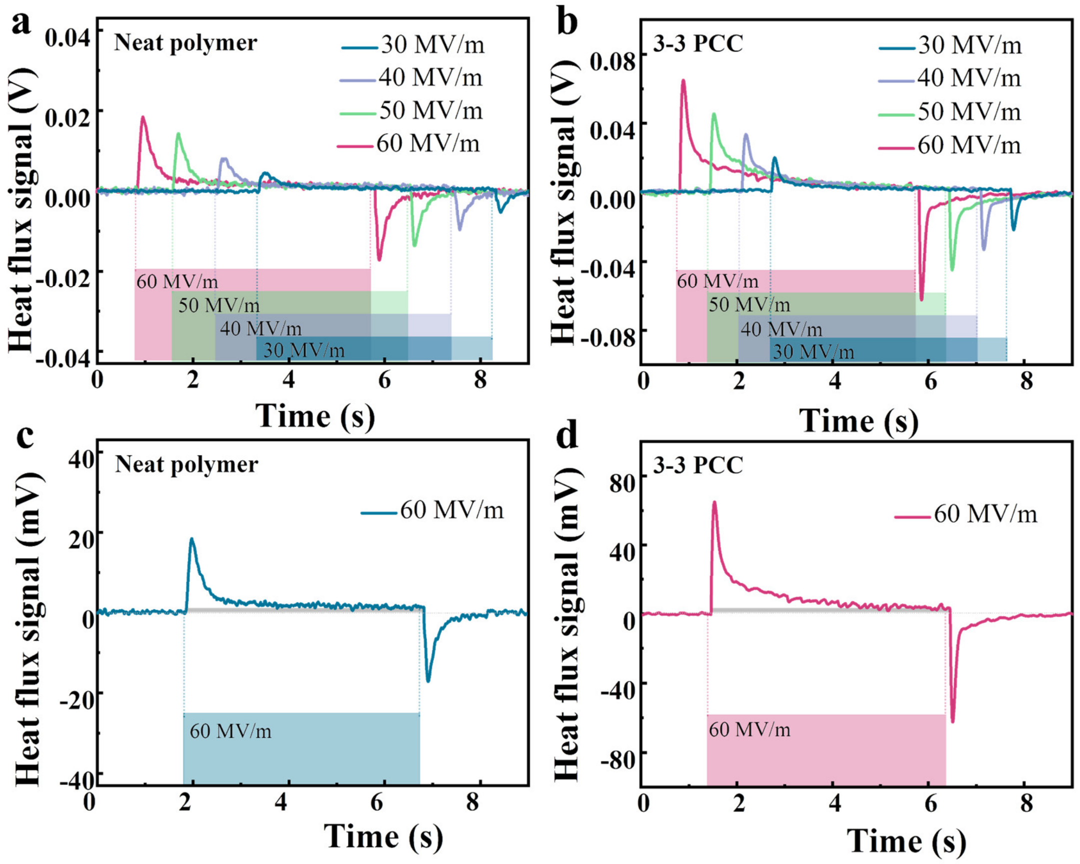

Supplementary Fig. 4. Comparison of electrocaloric-induced heat flux signals recorded in various electric fields. The exothermic and endothermic curves of **a**) neat polymer and **b**) 3-3 PCC samples with the applied and withdrawn electric fields. The electrocaloric and Joule heat-induced heat fluxes of **c**) neat polymer and **d**) 3-3 PCC samples under an electric field of 60 MV/m.

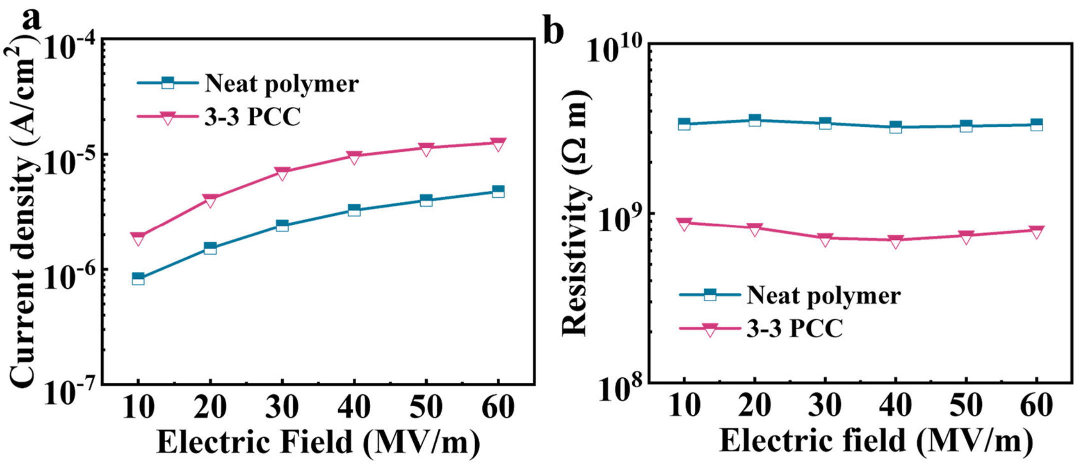

Supplementary Fig. 5. Comparison of resistivity and leakage current performance. The **a**) leakage current density and **b**) resistivity of the neat polymer and 3-3 PCC as a function of applied electric field.

Supplementary Fig. 4a and **b** show the exothermic and endothermic curves of Neat polymer and 3-3 PCC samples with the applied and withdrawn electric fields. In addition, **Supplementary Fig. 4c** and **d** show the electrocaloric and Joule heat-induced heat flux signals of Neat polymer and 3-3 PCC samples under an electric field of 60 MV/m recorded by the heat flux sensor. For example, the maximum heat flux signal induced by the electrocaloric effect at 3-3 PCC under an electric field of 60 MV/m is about 65.4 mV. The heat flux signal induced by Joule heating is only 5.6 mV (grey shaded area). This indicates that the Joule heat induced by the leakage of 3-3 PCC at 60 MV/m is small and almost negligible for the measurement of the electrocaloric performance. Furthermore, it is also shown that the leakage current of the 3-3 PCC composite at an electric field of 60 MV/m is small.

Additionally, **Supplementary Fig. 5** show the leakage current density and resistivity of pure polymer and 3-3 PCC under the action of electric field. As shown in **Supplementary Fig. 5a**, although the leakage current density increases with increasing electric field. However, the resistivity of neat polymer and 3-3 PCC remains relatively constant as the applied electric field increases (**Supplementary Fig. 5b**). This indicates that the leakage pathway does not increase and expand significantly with increasing applied electric field.

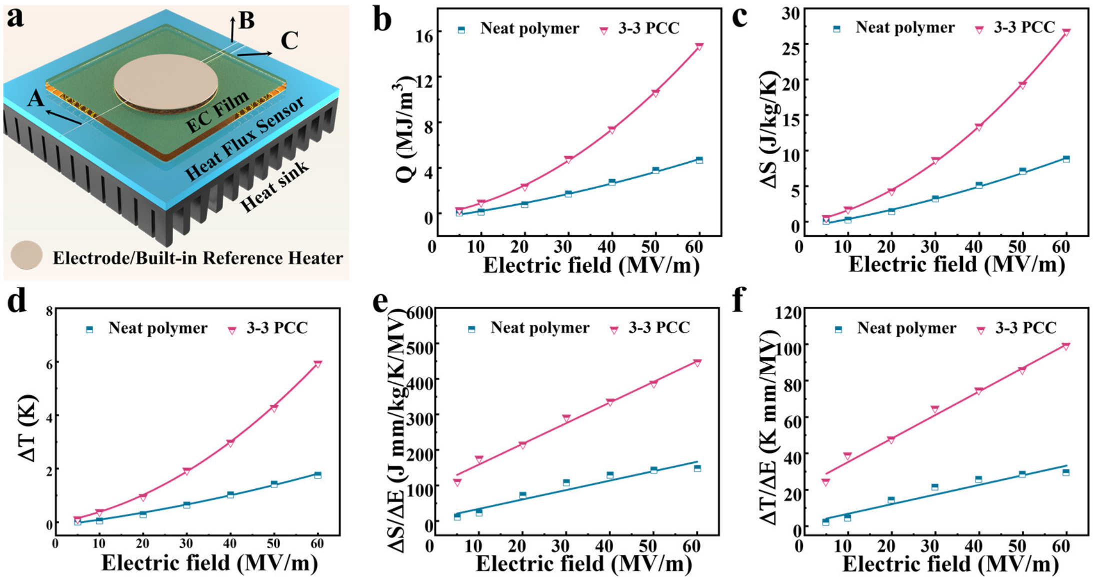

Supplementary Fig. 6. The excellent electrocaloric performance of 3-3 PCC materials. **a**) Schematic of the electrocaloric effect measurement with *in situ* calibration. **b**) Q, **c**) ΔS, **d**) ΔT and **e**) EC response ΔT / ΔE and **f**) ΔS / ΔE of the neat polymer and 3-3 PCC as a function of applied electric field.

we characterize the electrocaloric effect of the sample using an *in situ* calibrated measurement system. The test part from bottom to top is radiator, heat flux sensor, bottom reference heat resistance/electrode, electroactive film, and top reference heat resistance/electrode, respectively (**Supplementary Fig. 6a**). Firstly, a small voltage is applied on the top reference heat resistance to generate the known heat (*via* the two leads of A and B). The signal is detected by the heat flow sensor. Next, the *in situ* calibration is completed by constructing the relationship between the peak area of heat flow signal and the known heat Q_R. Finally, a high voltage is applied to the electrocaloric film (*via* the two leads of B and C); and the isothermal cooling energy density (Q) is obtained by integrating the peak area of the heat flow signal by the amount of heat per unit area (**Supplementary Fig. 4**). The detailed test process is referred to these works [1,2].

Supplementary Note 4. Relaxor ferroelectric behavior and dielectric properties of 3-3 PCC materials

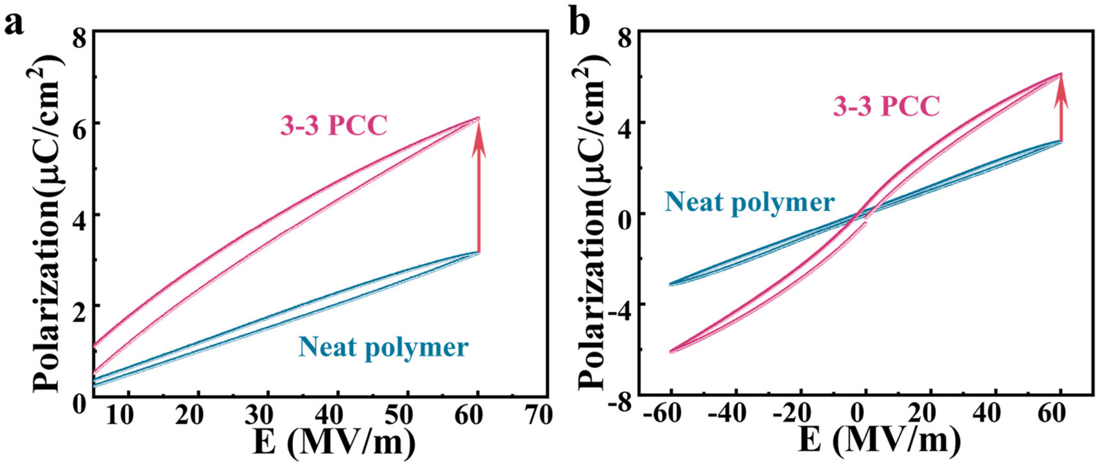

Supplementary Fig. 7. The polarization-electric field (P-E) loops of the terpolymer (Neat polymer) and 3-3 PCC: a) unipolar and b) bipolar P-E curves.

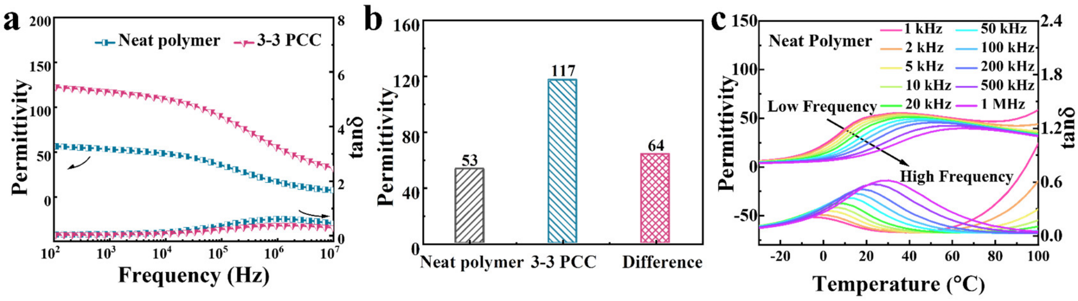

Supplementary Fig. 8. 3-D thermal conductivity network enhanced dielectric performances. **a**) The frequency dependence of the dielectric properties and **b**) dielectric constant at a frequency of 1 kHz for the neat polymer and 3-3 PCC. **c**)The temperature dependence of the dielectric permittivity and loss (*tanδ*) of the neat polymer.

The enhancement of polarization of the system is further verified by ferroelectric test (**Supplementary Fig. 7, Fig. 1f**). The polarization level of the 3-3 PCC is 6.08 μC/cm^2 at 60 MV/m, which is 193 % higher than that of the neat polymer (3.15 μC/cm^2). At lower electric field (10 MV/m), the polarization of the 3-3 PCC is 1.53 μC/cm^2, which is 258 % higher than that of the neat polymer (0.53 μC/cm^2).The frequency-dependent dielectric properties of the neat polymer and 3-3 PCC are plotted in **Supplementary Fig. 8a**. The corresponding histogram of the relative permittivity at 1 kHz is shown in **Supplementary Fig. 8b**. The dielectric constant of the 3-3 PCC is 117, which is 120% higher than that of the neat polymer (53). The three-dimensional network is successfully constructed in the polymer. The dielectric constants of the composites can be greatly increased when the filler constitutes an inter-transmission structure [3].

Supplementary Note 5. Structural origins of large electrocaloric performance

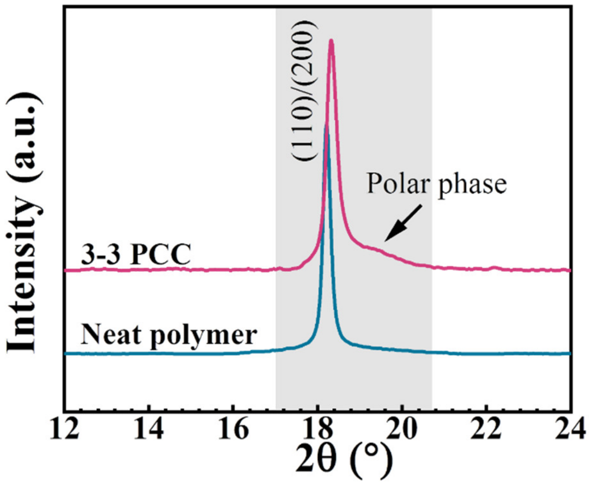

Supplementary Fig. 9. Analysis of crystal structures. X-ray diffraction pattern of the 3-3 PCC compared to the neat polymer.

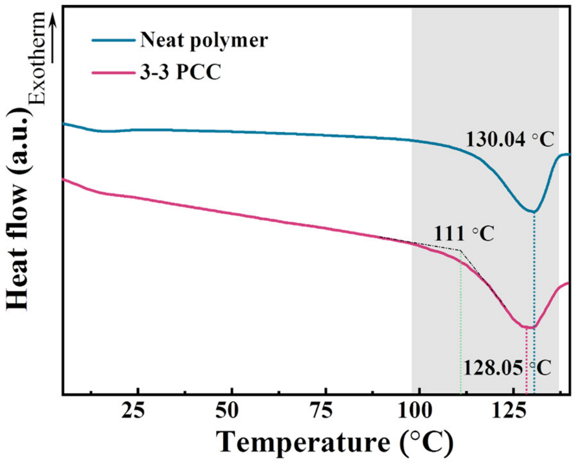

Supplementary Fig. 10. Melting behavior. The differential scanning calorimetry (DSC) curves of the terpolymer (neat polymer) and 3-3 PCC.

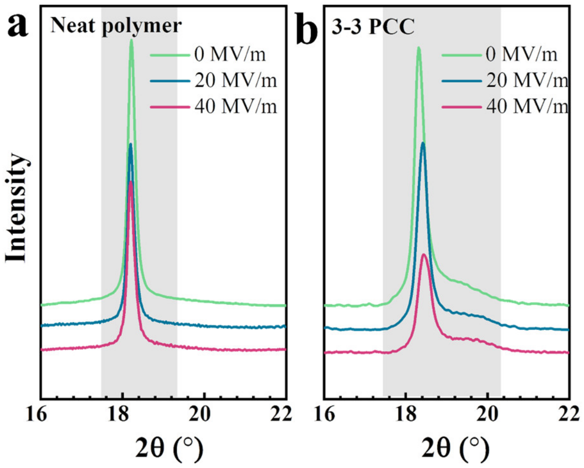

Supplementary Fig. 11. In situ X-ray diffractometer (XRD) to study the evolution of crystalline structure with electric field. In situ XRD patterns of the a) neat polymer and b) 3-3 PCC with increasing electric field.

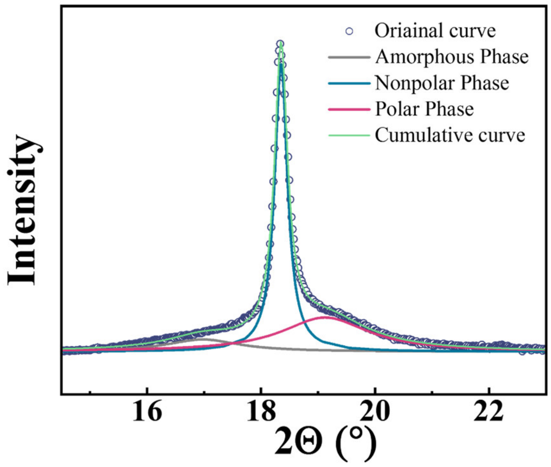

Supplementary Fig. 12. Structural origins of enhancing electrocaloric performance by 3-D thermally conductive networks. Estimation of the fractions of the non-polar and polar phases.

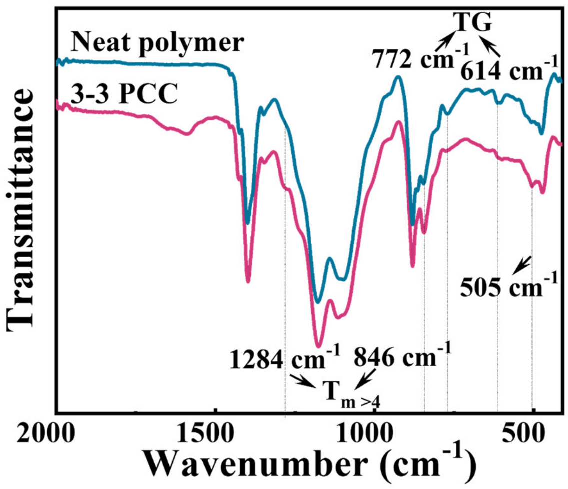

Supplementary Fig. 13 Conformational analysis. Fourier transform infrared (FTIR) spectrogram for the neat polymer and 3-3 PCC.

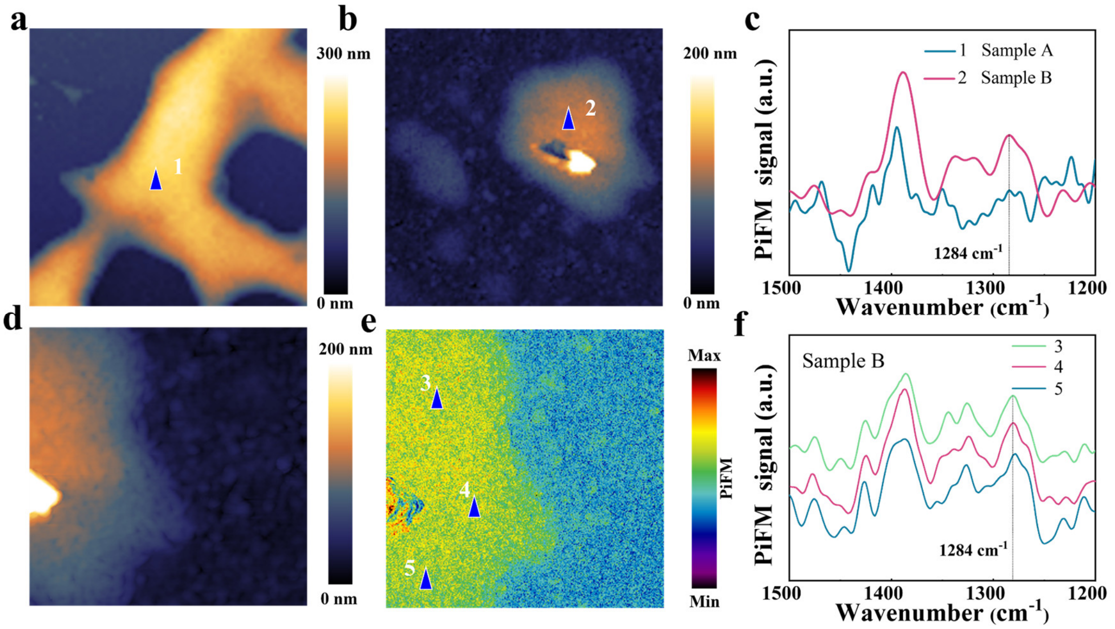

Supplementary Fig. 14. The topography and chemical composition maps of the polymer samples with different interface environment. AFM topography images (4.5×4.5 μm^2) of a) the sample A (Si/SiO_2/P(VDF-TrFE-CFE)) and b) sample B (Si/SiO_2/BCZT/P(VDF-TrFE-CFE)). c) PiFM point spectra for two kinds of polymer samples. d) AFM height image (2×2 μm^2) of the sample B and corresponding PiFM image taken at 1284 cm^{-1}. f) PiFM spectra at the points marked in **Supplementary Fig. 14e**.

We investigate the structural origin of the large electrocaloric properties of the 3-3 PCC. The evolution of phase structure after the 3-D CNet intercalation into polymers is analyzed by XRD. The low-angle diffraction peak from nonpolar phase becomes broader in the 3-3 PCC compared to that of the neat polymer (**Supplementary Fig. 9**). The crystal size is calculated using Scherer's equation. Smaller crystallite size of polymer (25 nm, Neat polymer: 32 nm) is realized in the 3-3 PCC, which is consistent with the DSC results (**Supplementary Fig. 10**). The corresponding enthalpies of melting are 19.05 and 20.83 J g^{-1} for the neat polymer and 3-3 PCC, respectively, indicating that the incorporation of the 3-D CNet increases the crystallinity of the polymer. Both reduced crystal size and increased crystallinity contribute to enhanced polarization of the polymer. This is because the enhancement in the crystallinity provides a higher polarizability, and the dipoles in smaller crystals are more easily aligned by the applied electric field. The experimental results further verify the

existence of polar phase in the 3-3 PCC (**Supplementary Fig. 9**). A new diffraction peak associated with the polar phase is observed at 2Θ of 19.8° in the 3-3 PCC at zero field strength [4].

We further demonstrate that the presence of polar nanodomains in 3-3 PCC at zero field strength can significantly reduce the potential barrier for nucleation growth of polar phases. The changes in the polar phase of the neat polymer and 3-3 PCC with increasing electric field are investigated using in situ XRD (**Supplementary Fig. 11**).

The percentage of polar and non-polar phases in the crystalline volume was calculated by the following formula:

$$f_n = \frac{A_n}{A_n + A_p} \qquad \text{(Eq. S1)}$$

$$f_p = \frac{A_p}{A_n + A_p} \qquad \text{(Eq. S2)}$$

The A_n is the integration areas of the non-polar phase and A_p is the integration area of the polar phase (**Supplementary Fig. 12**).

In the pure polymer, only a slight decrease in polar phase is detected with increasing electric field; and almost no increase in polar phase diffraction peaks is noticed (**Supplementary Fig. 11a**). In contrast, in the 3-3 PCC, as the electric field increases, the diffraction peaks of the non-polar phase progressively decrease, while the diffraction peaks of the polar phase gradually increase (**Supplementary Fig. 11b**). At the same time, both diffraction peaks are shifted to high angles with the increase of electric field. Quantitatively, the 3-3 PCC shows that volume fraction of the polar phase increases from the initial 32% to 43% when the electric field is increased to 40 MV m^{-1}, while for the pure polymer, the volume fraction is only 3.5% (**Fig. 2f**). Thus, the enhanced electrocaloric effect of the composite at low fields can be ascribed to the increased polar phases of the polymer in the presence of 3-D CNet. In addition, in the FTIR spectra, there is significant enhancement of absorption peaks at 846 and 1284 cm^{-1}, which are associated with the polar phase [1] (**Supplementary Fig. 13**). It also confirms that the polar phase can be induced in the 3-3 PCC.

In order to characterize that the introduction of 3-D CNet can induce the formation of polar nanodomains near the interface, we prepared two kinds of the polymer samples with different interface environment (**Supplementary Note 1**, Sample A: P(VDF-TrFE-CFE)/SiO_2/Si, Sample B: P(VDF-TrFE-CFE)/BCZT/SiO_2/Si). It can be seen that

the interfacial environment of the polymer in Sample B is similar to that of the polymer at the constructed 3-D CNet interface. The topography and chemical composition maps were simultaneously obtained using photo-induced force microscopy (**Supplementary Fig. 14**). Photo-induced force microscopy (PiFM) is a scan probe technique that offers images with spectroscopic contrast at a spatial resolution in the nanometer range.

As shown in **Supplementary Fig. 14a-c**, the polymer chains located at the BCZT ceramic interface still exhibit a stronger polar phase conformation at the nanometer range (significant enhancement of the characteristic peak at 1284 cm^{-1} associated with the polar phase). This suggests that the introduction of 3-D CNet into the polymer can induce the formation of polar nano-regions. **Supplementary Fig. 14d** and **Supplementary Fig. 14e** show atomic force microscope (AFM) height image (2×2 μm^2) of the polymer located at the BCZT ceramic interface and corresponding PiFM image taken at 1284 cm^{-1}. The polar nanodomains are uniformly distributed in the interfacial region, which means the formation of continuous polar domains, and the corresponding PiFM spectra show the same characteristics (**Supplementary Fig. 14f**). The above results provide direct evidence that polar nanodomains can be induced by the 3-D CNet at the interfacial region.

Supplementary Note 6. Heat transfer process simulated by finite element methods

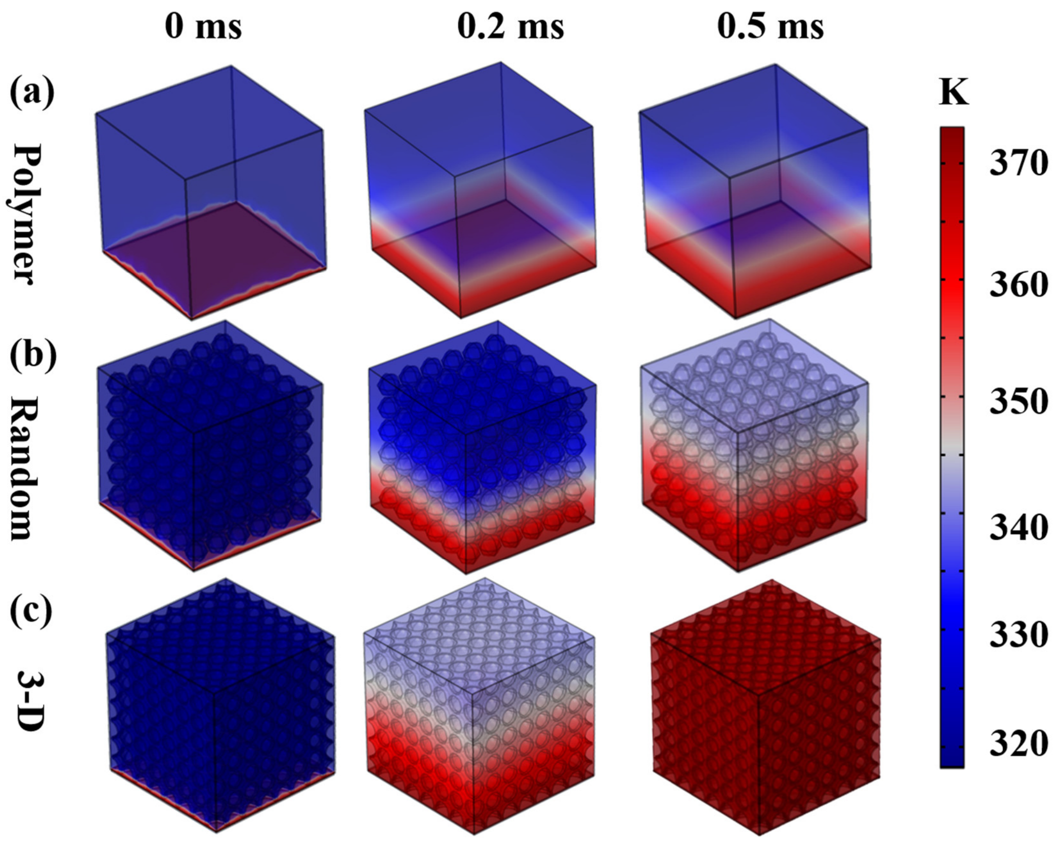

Supplementary Fig. 15. Heat transfer process simulated by finite element methods. Temperature distribution of the (a) neat polymer, (b) randomly distributed BCZT/polymer composite with BCZT volume ratio of 18%, (c) 3-D CNets/composite with 3-D CNets volume ratio of 18%.

Supplementary Note 7. An active electrocaloric cooling devices for chip thermal management

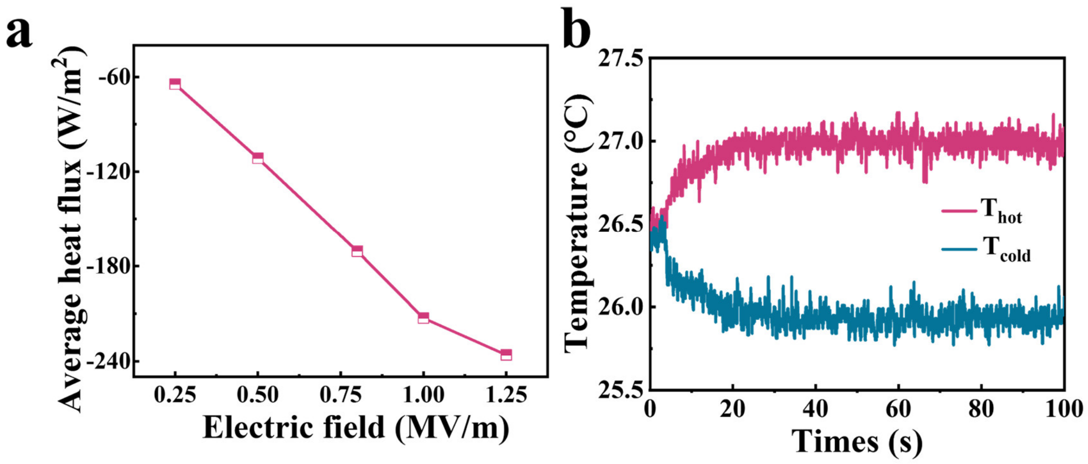

Supplementary Fig. 16. Cooling performance of the electrocaloric cooler. a) Frequency dependence of the average heat flux at an electric field of 30 MV/m. **b**) Temperature difference of electrocaloric cooling device at an electric field of 30 MV/m and a frequency of 1 Hz.

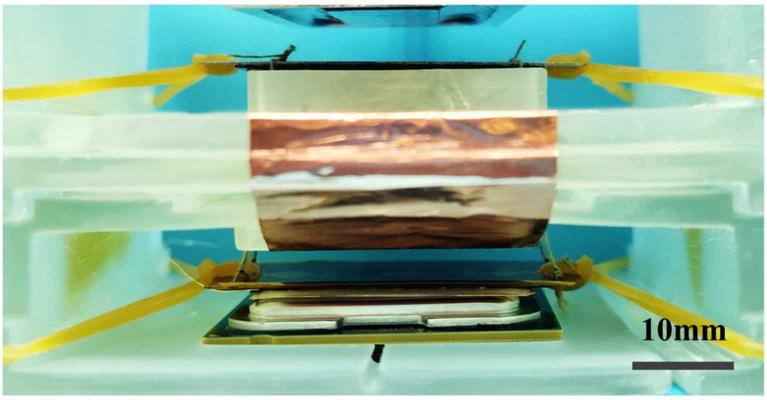

Supplementary Fig. 17. Optical image of the electrocaloric cooler for chip thermal management. Schematic diagram of an active electrocaloric device placed on top of the heating chip to evaluate its cooling performance

Supplementary Note 8. Infrared thermal image of CPU under different cooling conditions

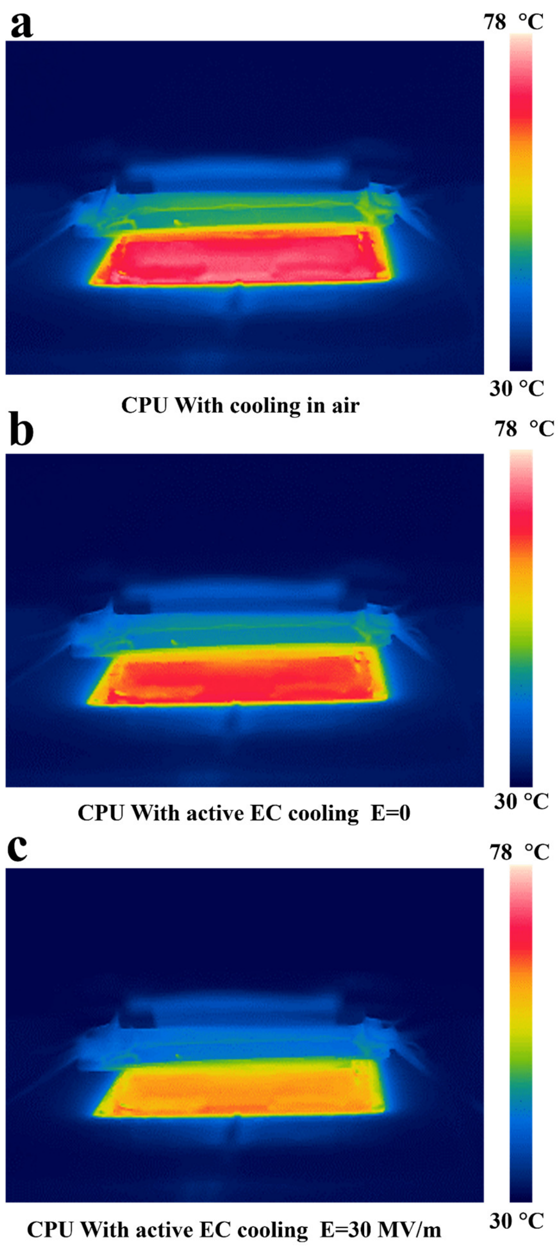

Supplementary Fig. 18. Infrared thermal image of central processing unit (CPU) under different cooling conditions: a) CPU with cooling in air, **b**) CPU with active electrocaloric (EC) cooling (E=0) and **c**) CPU with active EC cooling (E=30 MV/m)

Supplementary References

1. Qian, X. S. *et al.* High-entropy polymer produces a giant electrocaloric effect at low fields. *Nature* **600**, 664-669 (2021).
2. Shi, J. *et al.* Electrocaloric Cooling Materials and Devices for Zero-Global-Warming-Potential, High-Efficiency Refrigeration. *Joule* **3**, 1200-1225 (2019).
3. Luo, S. *et al.* Construction of a 3D-BaTiO3 network leading to significantly enhanced dielectric permittivity and energy storage density of polymer composites. *Energy Environ. Sci.* **10**, 137-144 (2017).
4. Bao, H. M. *et al.* Phase transitions and ferroelectric relaxor behavior in P(VDF-TrFE-CFE) terpolymers. *Macromolecules* **40**, 2371-2379 (2007).
