# Supplementary material for: Thermal management of chips by a device prototype using synergistic effects of 3-D heat-conductive network and electrocaloric refrigeration
Source: Nat Commun. 2022 Oct 4;13:5849. doi: 10.1038/s41467-022-33596-z (PMC9532434; doi:10.1038/s41467-022-33596-z)
Supplement: Supplementary file 3 — Description of Additional Supplementary Files [file 41467_2022_33596_MOESM3_ESM.pdf]

Description of Additional Supplementary Files

Supplementary Movie 1
The movie of the dynamic evolution of temperature versus time during heating and cooling.

Supplementary Movie 2
The movie of electrocaloric cooling device operating at low frequency (1 Hz).

Supplementary Movie 3
The movie of electrocaloric cooling device operating at low frequency (5 Hz).
