# Supplementary material for: Thermal management of chips by a device prototype using synergistic effects of 3-D heat-conductive network and electrocaloric refrigeration
Source: Nat Commun. 2022 Oct 4;13:5849. doi: 10.1038/s41467-022-33596-z (PMC9532434; doi:10.1038/s41467-022-33596-z)
Supplement: Supplementary file 2 — Peer Review File [file 41467_2022_33596_MOESM2_ESM.pdf]

nature portfolio

Peer Review File

Thermal Management of Chips by a Device Prototype Using Synergistic Effects of 3-D Heat-Conductive Network and Electrocaloric Refrigeration

Open Access This file is licensed under a Creative Commons Attribution 4.0 International License, which permits use, sharing, adaptation, distribution and reproduction in any medium or format, as long as you give appropriate credit to the original author(s) and the source, provide a link to the Creative Commons license, and indicate if changes were made. In the cases where the authors are anonymous, such as is the case for the reports of anonymous peer reviewers, author attribution should be to 'Anonymous Referee' followed by a clear attribution to the source work. The images or other third party material in this file are included in the article's Creative Commons license, unless indicated otherwise in a credit line to the material. If material is not included in the article's Creative Commons license and your intended use is not permitted by statutory regulation or exceeds the permitted use, you will need to obtain permission directly from the copyright holder. To view a copy of this license, visit http://creativecommons.org/licenses/by/4.0/.

REVIEWER COMMENTS

Reviewer #1 (Remarks to the Author):

This work reports a ferroelectric composite material for high electrocaloric (ECE) property. Different from the conventional composites that combining the advances of different components with a 0-3 structure, the composite of this work consists of 3-D interconnected ferroelectric ceramics embedded in a polymer matrix. The interconnected 3-D ceramic skeletons not only induce high polarization and strong ECE of the material, but also construct a thermal conductive network for high thermal conductivity. Furthermore, the designed composite has been successfully equipped on a prototype cooling device derived by the electromagnetic mechanism. The concerns are listed as below.
(1) As seen from Fig. 1a,b, Fig. 2a and Supplementary Figure S2, the ceramic skeletons are embedded in the ferroelectric polymers with a 3-D interconnected structure. However, as stated in “Note 1. Experimental Section”, “3-3 polymer/ceramic composite (abbreviated as 3-3 PCC) materials were obtained by converting aforementioned materials to a dense body through hot-pressing at 140 °C for 10 min, under a pressure of 7 MPa.” In my understanding, during the hot-pressing process, the thickness of the 3-D composites would be decreased significantly form several millimeters to less than 1 millimeters (this was confirmed by the authors as they stated that “The volume of the active area of a typical electrocaloric stack is 25*20*0.15 mm”, which means the thickness of the pressed films is 150 microns), and the 3-D structure of the ceramic skeleton would be crashed and damaged. In this case, the microstructure of the material seems to be inconsistent with the strategy and the simulation model (Figure 2a and Supplementary Figure S12) designed by the authors. Another concern is that if the 3-d structure of the ceramic is maintained in the composites, the thickness of the sample should be larger than 1 mm, and therefore a very high voltage has to be applied on the sample for the ECE. How the authors deal with this issue?
(2) As the ECE was chartered by a heat flux sensor, the endothermal and exothermal curves with the application and withdrawing of electric fields are suggested to be provided to evaluate the joule heating, and the joule heating of the samples should be discussed.
(3) Can the prepared 3-D composites be operated under higher electric fields for stronger ECE? Or the leakage current increases significantly with the increased field? The resistivity as a function of electric fields are suggested to be provided.
(4) More data for the cooling device should be shown. For example, without a CPU, what the temperature difference can be obtained from the hot side and cold side of the device? How about the relation between the temperature difference and the thermal conductivity/thermal mass of thermal loads of the device? The performance of the device operated with various frequencies and electric fields are also suggested to be tested.

Reviewer #2 (Remarks to the Author):

Li and Shen et al reported the thermal conductivity and electric refrigeration performance of a relaxor-type ferroelectric 3-3 P(VDF-TrFE-CFE)/BCZT composites. By using XRD, DSC, and FTIR, the author indicates the introduction of BCZT can effectively change the crystallinity and the content of polar phase of P(VDF-TrFE-CFE). Moreover, dielectric properties and P-E curves are performed to compare the improvement of polarization in P(VDF-TrFE-CFE). And by using a sensor plus to the calculation from the formula in Page 6, line 137, the author has the conclusions of a 206% increase in the electrocaloric performance and a 300% enhancement in the thermal conductivity of in this kind of material

compared to that of pure polymer. Then the authors believe this electrocaloric composite and electromagnetic actuation mechanism have the prospect of application in a single heat spot cooling of 5G chip.
In the referee’s opinion, this work is well organized and is recommended to publish on this famous journal after minor revision. The following questions are for ther author to improve the quality of this manuscript.
1) Why the annealed temperature in experimental section is exactly 106°C, and what is the particular value of this point?
2) This material is actually a relaxor ferroelectrics, and therefore the “ferroelectric” in note4 is improper. Moreover, the unipolar P-E curve cannot show the ferroelectric property of this composite.
3) Why not provide the temperature vs dielectric permittivity spectrum of the 3-3 PCC for comparing to neat P(VDF-TrFE-CFE) to illustrate the differences.

Reviewer #3 (Remarks to the Author):

Authors have constructed a 3D continuous network in BCZT based ferroelectric composites, where structural design, microstructure analysis, dielectric property and electrocaloric effect performance through a configurated device are all discussed. I would say this kind of structural design is quite interesting despite more information/details about the approach as required. Moreover, the discussion of polar nanoregions shall be improved/strengthened in revealing their effect on the change of polar phase. On the other hand, the designed device (electrocaloric cooler) in demonstrating the electrocaloric performance is very important considering its effectiveness and reproducibility for the refrigeration applications. Thus, I have some concerns for the current version of this work, and my comments are included as below.
1. Abstract part shall be substantially improved, where key challenge, solution, conceptual innovation, key result and significant advances of this work shall be included. However, current version of the abstract is a little bit long, messy and shall be shortened to reveal the significant innovations and advances of this work.
2. Experimental part: It is very strange that substance BN seems to be missing during the description of experimental details. However, BN is playing a predominant role in affecting the conductivity of fabricated composites.
3. As mentioned by authors, introduction of 3-D CNet into the polymer can induce the formation of polymer nano-regions, but there is no direct evidence to support this claim. XRD results only show changes in the polar phase which might be induced by the claimed polymer nanodomains. So can authors please provide direct evidence to reveal the formed polar nanodomains induced by the constructed 3-D CNet. I would suggest using conductive force microscope technique to give it a try.
4. One more question, volume fraction of polar phase has been analyzed in the 3-3 PCC as a function of the applied electric field. However, how to determine the specific volume fraction of polar phase? Some main equations or crucial calculation steps shall be provided in the supplementary file to make readers easier check the repeatability of the results.
5. Can authors explain the reasonability of device design in this work? As a number of components including electromagnetic drive and heat flux are used in the design, thus, how to make sure the effectiveness of this device in demonstrating the electrocaloric effect performance becomes very important.
6. For the constructed 3D continuous network as claimed, can authors provide more information to clearly explain how to exactly achieve this kind of 3D structural design? I

would like to know more details about this and then evaluate the effective/feasibility of this method. For the moment, BN is missing in the experiment part, and more details of this approach in achieving 3D network are required to guarantee the reproducibility of this method.

7. The electrocaloric cooler can only successfully function below 80 thermo degrees, otherwise high risk will emerge. Thus, I would like to know whether authors have figured out any other option or solution in improving this kind of sequence for the future work. One more serious concern is how to make sure the device only function below this certain temperature point, as it is very easy to reach 80 thermo degrees under practical circumstances.

Point-by-point response to the reviewers' comments:

Reviewer #1

Comments:

This work reports a ferroelectric composite material for high electrocaloric (ECE) property. Different from the conventional composites that combining the advances of different components with a 0-3 structure, the composite of this work consists of 3-D interconnected ferroelectric ceramics embedded in a polymer matrix. The interconnected 3-D ceramic skeletons not only induce high polarization and strong ECE of the material, but also construct a thermal conductive network for high thermal conductivity. Furthermore, the designed composite has been successfully equipped on a prototype cooling device derived by the electromagnetic mechanism. The concerns are listed as below.

Comment 1. As seen from Fig. 1a, b, Fig. 2a and Supplementary Figure S2, the ceramic skeletons are embedded in the ferroelectric polymers with a 3-D interconnected structure. However, as stated in "Note 1. Experimental Section", "3-3 polymer/ceramic composite (abbreviated as 3-3 PCC) materials were obtained by converting aforementioned materials to a dense body through hot-pressing at 140 °C for 10 min, under a pressure of 0.7 MPa." In my understanding, during the hot-pressing process, the thickness of the 3-D composites would be decreased significantly form several millimeters to less than 1 millimeters (this was confirmed by the authors as they stated that "The volume of the active area of a typical electrocaloric stack is 25*20*0.15 mm", which means the thickness of the pressed films is 150 microns), and the 3-D structure of the ceramic skeleton would be crashed and damaged. In this case, the microstructure of the material seems to be inconsistent with the strategy and the simulation model (Figure 2a and Supplementary Figure S12) designed by the authors. Another concern is that if the 3-d structure of the ceramic is maintained in the composites, the thickness of the sample should be larger than 1 mm, and therefore a very high voltage has to be applied on the sample for the ECE. How the authors deal with this issue?

Answer 1: Thank you for pointing out this important issue. Embedding 3-D ceramic

networks into polymer matrices and ensuring their high quality is a great challenge. In order to ensure that the continuous 3-D network is successfully introduced into the polymer, the following details need to be taken into account. First, when filling the P(VDF-TrFE-CFE) into the 3-D CNet, it is important to repeat the process several times to ensure that the 3-D CNet is completely filled with the polymer matrix. Further, it is important to control the pressure not to be too high during the hot-pressing process. In addition, during the hot-pressing process, we place a pure film of 100 μm thickness on the top and bottom side of the hot-pressing precursor as a buffer layer. Typical thickness after hot-pressing is around 800 μm. In order to test the electrical properties and reduce the applied voltage, the samples are thinned by abrasive paper to achieve the desired thickness.

In addition, we have added the relevant discussion from the experimental section to address this issue, with corrections as follows. *Note 1. Experimental Section: "Next, 3-3 polymer/ceramic composite (abbreviated as 3-3 PCC) materials were obtained by converting aforementioned materials to a dense body through hot-pressing at 140 °C for 10 min, under a pressure of 0.7 MPa. A 100-μm thick P(VDF-TrFE-CFE) film was placed on the top and bottom surfaces of the hot-pressed precursor as a buffer layer. The typical thickness after hot-pressing was around 800 μm. Subsequently, the samples were thinned using abrasive paper to achieve the desired thickness. Finally, the resulting 3-3 PCC films were annealed in a vacuum oven at 106 °C for 10 h to improve the crystallinity of the films."*

Comment 2. As the ECE was chartered by a heat flux sensor, the endothermal and exothermal curves with the application and withdrawing of electric fields are suggested to be provided to evaluate the joule heating, and the joule heating of the samples should be discussed.

Answer 2: Thank you very much for your valuable suggestions. **Figure RI-1a** and **b** show the exothermic and endothermic curves of the neat polymer and 3-3 PCC samples with the applied and withdrawn electric fields. In addition, **Figure RI-1c** and **d** show the electrocaloric and Joule heat-induced heat flux signals of the neat polymer and 3-3 PCC samples under an electric field of 60 MV/m recorded by the heat flux sensor. For

example, the maximum heat flux signal induced by the electrocaloric effect of 3-3 PCC under an electric field of 60 MV/m is about 65.4 mV. The heat flux signal induced by joule heating is only 5.6 mV (grey shaded area). Thus, the joule heat induced by the leakage of 3-3 PCC at 60 MV/m is small and almost negligible for the measurement of the electrocaloric performance. Furthermore, the leakage current of the 3-3 PCC composite at an electric field of 60 MV/m is small. This is further demonstrated by the resistivity variation relationship with increasing electric field (**Figure RI-4**).

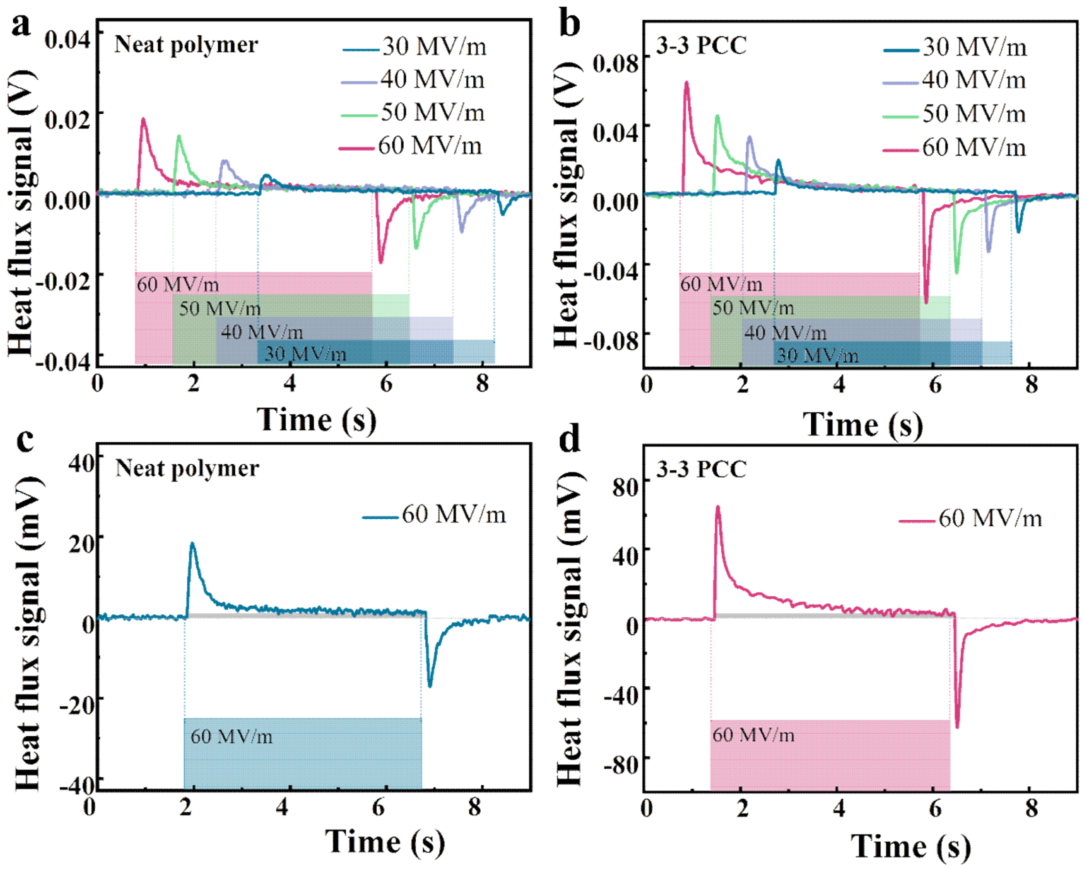

Figure RI-1. The exothermic and endothermic curves of **a**) neat polymer and **b**) 3-3 PCC samples with the applied and withdrawn electric fields. The electrocaloric and Joule heat-induced heat fluxes of **c**) neat polymer and **d**) 3-3 PCC samples under an electric field of 60 MV/m.

Figure RI-1 is added in **Supplementary Figure S4.**

Comment 3. Can the prepared 3-D composites be operated under higher electric fields for stronger ECE? Or the leakage current increases significantly with the increased field? The resistivity as a function of electric fields are suggested to be provided.

Answer 3: Thanks for your valuable suggestions. Following your suggestion, stronger ECE is achieved in the prepared 3-D composites, as shown in **Figures RI-2** and **RI-3**. For instance, an isothermal cooling energy density Q up to 14.72 MJ m^{-3} with an electrocaloric strength $Q/\Delta E$ of 245.3 kJ m^{-2} MV^{-1} is achieved at 60 MV m^{-1}. Simultaneously, the 3-3 PCC at 60 MV m^{-1} displays a ΔS of 26.76 J kg^{-1} K^{-1}, a ΔT of 5.94 K, a ΔS/ΔE of 446 J mm kg^{-1} K^{-1} MV^{-1}, and a ΔT/ΔE of 99 K mm MV^{-1}, which is 240% higher than the state-of-the-art electrocaloric polymers at the same field strength. Additionally, **Figure RI-4** show the leakage current density and resistivity of the pure polymer and 3 - 3 PCC under the action of electric field. As shown in **Figure RI-4a**, although the leakage current density increases with increasing electric field, the resistivity of the neat polymer and 3-3 PCC remains relatively constant as the applied electric field increases (**Figure RI-4b**). This indicates that the leakage pathway does not increase and expand significantly with increasing electric field.

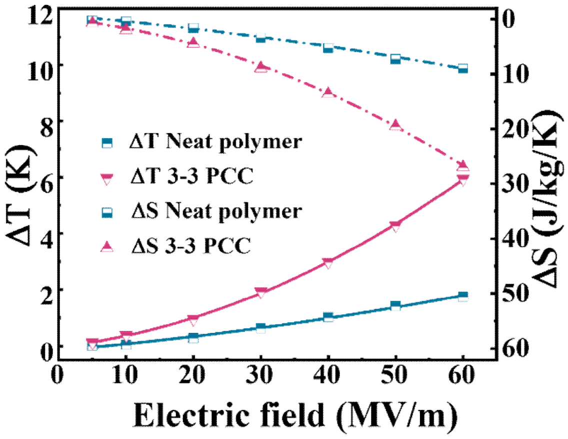

Figure RI-2. The ΔS and ΔT of the neat polymer and 3-3 PCC as a function of applied electric field.

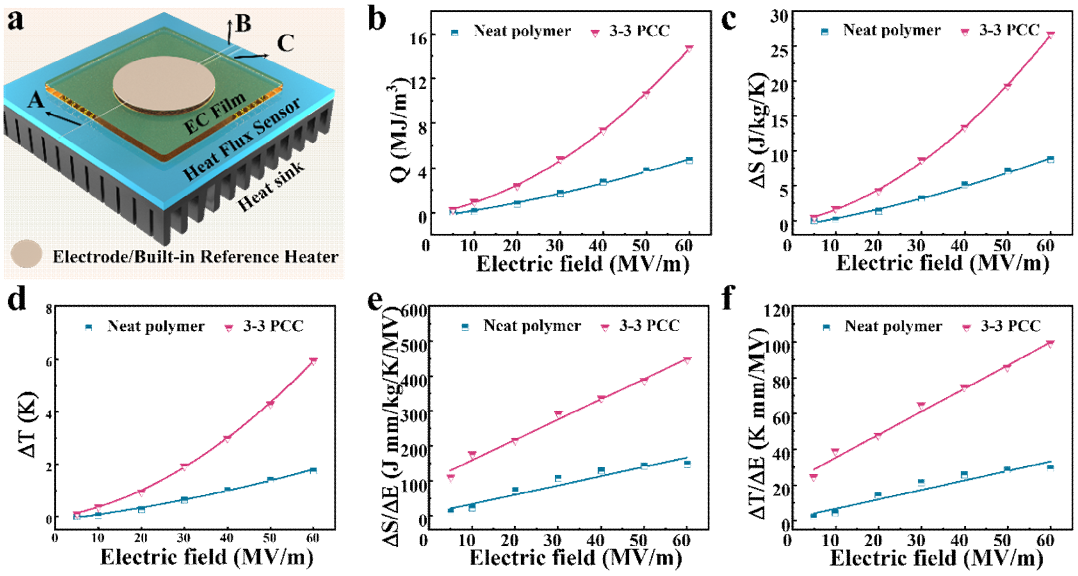

Figure RI-3. 3-D thermal conductivity network enhanced EC performances. **a**) Schematic of the ECE measurement with *in situ* calibration. **b**) Q, **c**) ΔS, **d**) ΔT and **e**) EC response ΔT / ΔE and **f**) ΔS / ΔE of the neat polymer and 3-3 PCC as a function of applied electric field.

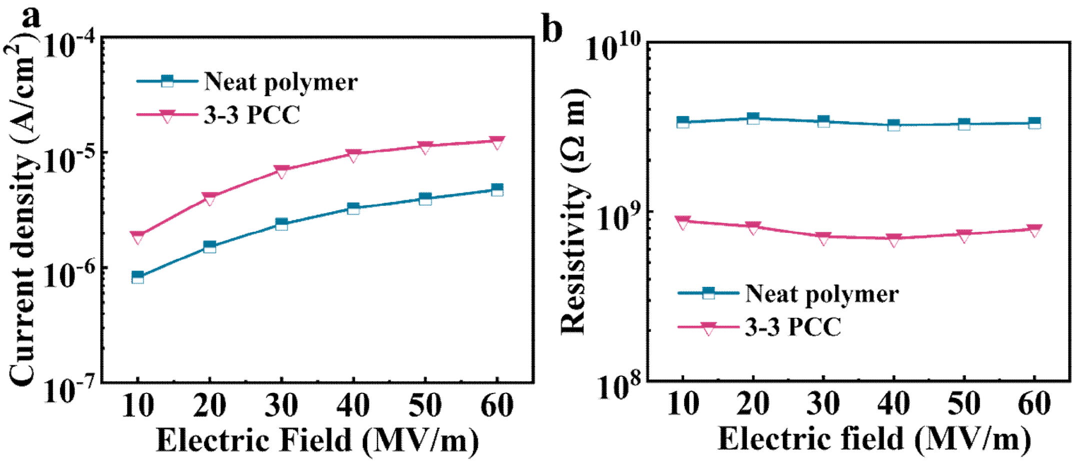

Figure RI-4. The a) leakage current density and b) resistivity of the neat polymer and 3-3 PCC as a function of applied electric field.

Figure RI-2 is added in **Figure 1e. Figures RI-3** and **RI-4** are added in Supplementary **Figures S5** and **S6**, respectively.

Comment 4. More data for the cooling device should be shown. For example, without

a CPU, what the temperature difference can be obtained from the hot side and cold side of the device? How about the relation between the temperature difference and the thermal conductivity/thermal mass of thermal loads of the device? The performance of the device operated with various frequencies and electric fields are also suggested to be tested.

Answer 4: Sincere thanks go out to you for the valuable suggestions. The maximum heat flux of the electrocaloric stack on the heating and cooling side versus the applied electric field is measured by a heat flux sensor at an operation frequency of 0.1 Hz (**Figure RI-5a**). The maximum heat fluxes for heating and cooling at the electric field of 30 MV m^{-1} are 288 and -272 W m^{-2}, respectively. **Figure RI-5b** shows the effect of operating frequency on the average heat flux. An average cooling heat flow of -213 W m^{-2} can be achieved at a frequency of 1 Hz. Furthermore, the average cooling heat flux can be further increased at higher frequencies (-236 W m^{-2} at a frequency of 1.25 Hz). Similarly, the ideal temperature span (1.1 K) of the electrocaloric cooling device is obtained at a frequency of 1 Hz with an electric field of 30 MV/m (**Figure RI-5c**).

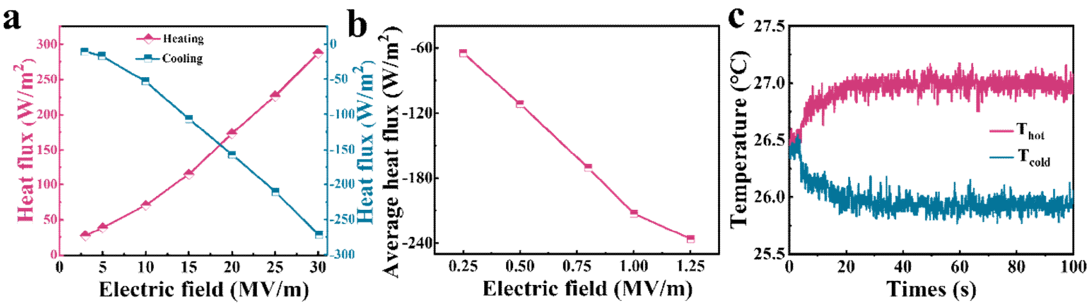

Figure RI-5. a) The maximum heat flux of the electrocaloric stack on the heating and cooling side versus the applied electric field is measured by a heat flux sensor at an operation frequency of 0.1 Hz. **b**) Frequency dependence of the average heat flux at an electric field of 30 MV/m. **c**) Temperature difference of electrocaloric cooling device at an electric field of 30 MV/m and a frequency of 1 Hz.

Figure RI-5a is shown in **Figure 3f. Figure RI-5b, c** is added in **Supplementary Figure S16.**

Reviewer #2:

Comments:

Li and Shen et al reported the thermal conductivity and electric refrigeration performance of a relaxor-type ferroelectric 3-3 P(VDF-TrFE-CFE)/BCZT composites. By using XRD, DSC, and FTIR, the author indicates the introduction of BCZT can effectively change the crystallinity and the content of polar phase of P(VDF-TrFE-CFE). Moreover, dielectric properties and P-E curves are performed to compare the improvement of polarization in P(VDF-TrFE-CFE). And by using a sensor plus to the calculation from the formula in Page 6, line 137, the author has the conclusions of a 206% increase in the electrocaloric performance and a 300% enhancement in the thermal conductivity of in this kind of material compared to that of pure polymer. Then the authors believe this electrocaloric composite and electromagnetic actuation mechanism have the prospect of application in a single heat spot cooling of 5G chip.

In the referee's opinion, this work is well organized and is recommended to publish on this famous journal after minor revision. The following questions are for their author to improve the quality of this manuscript.

Comment 1. Why the annealed temperature in experimental section is exactly 106°C, and what is the particular value of this point?

Answer 1: Thank you for taking up this important issue. 106 °C was chosen as the annealing temperature, because we have found by DSC measurements that the starting melting temperature of 3-3 PCC is 111 °C (**Figure RII-1**). This is the lowest temperature at which the crystalline zone is destroyed. In order to increase the crystallinity without destroying the microcrystalline zone, therefore, we chose to anneal at 5 °C lower than the starting melting temperature, i.e. 106 °C.

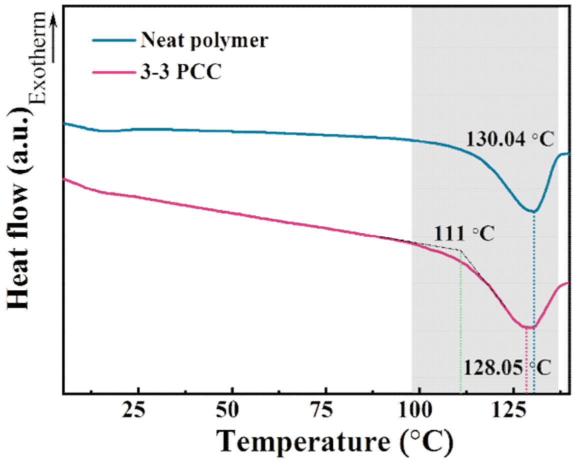

Figure RII-1. The DSC curves of the neat polymer and 3-3 PCC**.**

Figure RII-1 is shown in **Supplementary Figure S10.**

Comment 2. This material is actually a relaxor ferroelectrics, and therefore the "ferroelectric" in note4 is improper. Moreover, the unipolar P-E curve cannot show the ferroelectric property of this composite.

Answer 2: Thank you for your suggestion. Following your suggestion, we have changed the relevant statements. The bipolar P-E curve is added to the **Figure S7** of the revised Supplementary information **(Figure RII-2)**.

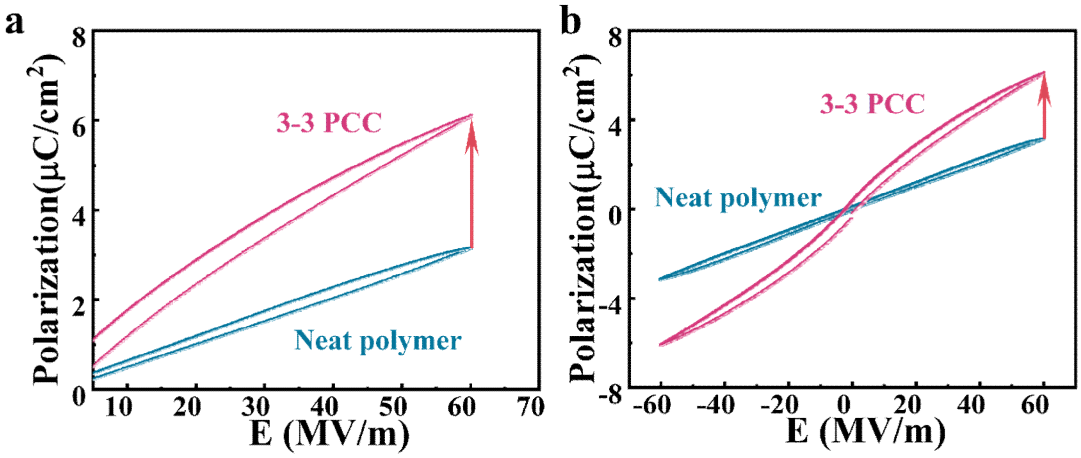

Figure RII-2. The polarization-electric field (P-E) loops of the terpolymer (the neat polymer) and 3-3 PCC: a) unipolar and b) bipolar P-E curves.

Figure RII-2 is shown in **Supplementary Figure S7.**

Comment 3. Why not provide the temperature vs dielectric permittivity spectrum of the 3-3 PCC for comparing to neat P(VDF-TrFE-CFE) to illustrate the differences.

Answer 3: Thanks for your kind suggestions. Compared to that of the neat P(VDF-TrFE-CFE), the temperature-dependent dielectric properties of the 3-3 PCC are discussed in the **Figure 1g** of the revised manuscript.

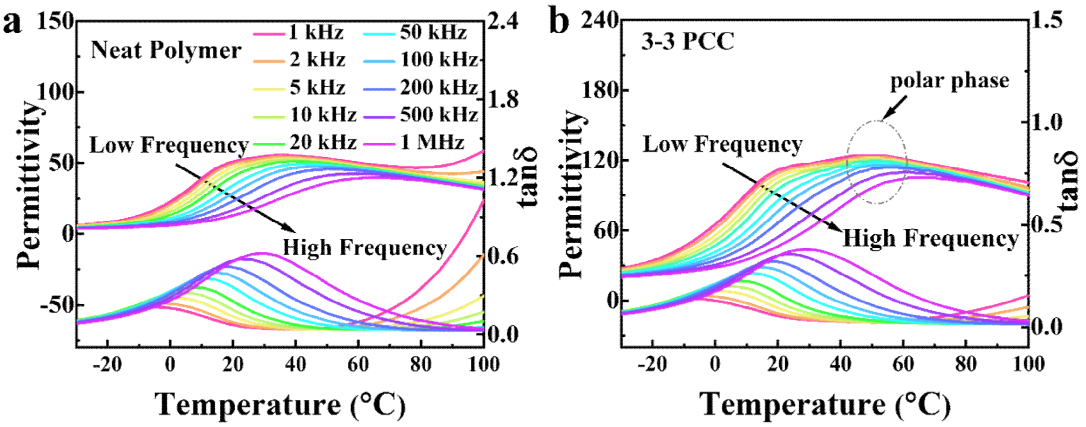

Figure RII-3. The temperature-dependence of the dielectric permittivity and loss (tanδ) of a) the neat polymer and b) 3-3 PCC.

Figure RII-2a is shown in Figure 1g. **Figure RII-2b** is shown in **Supplementary Figure S8c.**

Reviewer #3:

Comments:

Authors have constructed a 3D continuous network in BCZT based ferroelectric composites, where structural design, microstructure analysis, dielectric property and electrocaloric effect performance through a configurated device are all discussed. I would say this kind of structural design is quite interesting despite more information/details about the approach as required. Moreover, the discussion of polar nanoregions shall be improved/strengthened in revealing their effect on the change of polar phase. On the other hand, the designed device (electrocaloric cooler) in demonstrating the electrocaloric performance is very important considering its effectiveness and reproducibility for the refrigeration applications. Thus, I have some concerns for the current version of this work, and my comments are included as below.

Comment 1. Abstract part shall be substantially improved, where key challenge, solution, conceptual innovation, key result and significant advances of this work shall be included. However, current version of the abstract is a little bit long, messy and shall be shortened to reveal the significant innovations and advances of this work.

Answer 1: Thanks for the valuable suggestions. We have re-written the abstract accordingly:

With speeding up development of 5G chips, high-efficient thermal structure and precise management of tremendous heat becomes a substantial challenge to the power-hungry electronics. Thermal management has been achieved by reduction of thermal impedance, extraction of heat with the fluid in micro-channels, and electrocaloric refrigeration in solid-state materials with electric dipolar constituents. Here, we demonstrate an interpenetrating architecture of electrocaloric polymer with highly thermally conductive pathways that achieves a 240% increase in the electrocaloric performance and a 300% enhancement in the thermal conductivity of the polymer. A scaled-up version of the device prototype for a single heat spot cooling of 5G chip is fabricated utilizing this electrocaloric composite and electromagnetic actuation. The

continuous 3-D thermal conductive network embedded in the polymer acts as nucleation sites of the ordered dipoles under applied electric field, efficiently collects thermal energy at the hot-spots arising from field-driven dipolar entropy change, and opens up the high-speed conduction path of phonons. The synergy of two components, thus, tackles the challenge of sluggish heat dissipation of the electroactive polymers and their contact interfaces with low thermal conductivity, and more importantly, significantly reduces the electric energy for switching the dipolar states during the electrocaloric cycles, and increases the manipulable entropy at the low fields. Such a feasible solution is inevitable to the precisely fixed-point thermal management of next-generation smart microelectronic devices.

Comment 2. Experimental part: It is very strange that substance BN seems to be missing during the description of experimental details. However, BN is playing a predominant role in affecting the conductivity of fabricated composites.

Answer 2: We are very sorry for any misleading information in the description of the three-dimensional BN thermal conductivity network literature cited in the introduction section. we have re-written this part. In this work, we chose lead-free ferroelectric ceramic $Ba_{0.85}Ca_{0.15}Zr_{0.1}Ti_{0.9}O_3$ (BCZT) as a continuous 3-D CNet.

There is no double that BN is also a great choice, and we found 3-D BN composite has fairly good thermal conductivity and electric performance. We hope to share those results in near future.

Comment 3. As mentioned by authors, introduction of 3-D CNet into the polymer can induce the formation of polymer nano-regions, but there is no direct evidence to support this claim. XRD results only show changes in the polar phase which might be induced by the claimed polymer nanodomains. So can authors please provide direct evidence to reveal the formed polar nanodomains induced by the constructed 3-D CNet. I would suggest using conductive force microscope technique to give it a try.

Answer 3: Thanks very much for your helpful suggestions. The direct observation of

polar nanodomains is still a great challenge for the current state of the art. In order to characterize that the introduction of 3-D CNet can induce the formation of polar nanodomains near the interface, we prepared two kinds of the polymer samples with different interface environment (Sample A: P(VDF-TrFE-CFE)/SiO_2/Si, Sample B: P(VDF-TrFE-CFE)/BCZT/SiO_2/Si). It can be seen that the interfacial environment of the polymer in Sample B is similar to that of the polymer at the constructed 3-D CNet interface. The topography and chemical composition maps were simultaneously obtained using photo-induced force microscopy (**Figure RIII-1**). Photo-induced force microscopy (PiFM) is a scan probe technique that offers images with spectroscopic contrast at a spatial resolution in the nanometer range.

As shown in **Figure RIII-1a-c**, the polymer chains located at the BCZT ceramic interface still exhibit a stronger polar phase conformation at the nanometer range (significant enhancement of the characteristic peak at 1284 cm^{-1} associated with the polar phase). This suggests that the introduction of 3-D CNet into the polymer can induce the formation of polar nano-regions. **Figure RIII-1d** and **e** show atomic force microscope (AFM) height image (2 × 2 μm^2) of the polymer located at the BCZT ceramic interface and corresponding PiFM image taken at 1284 cm^{-1}. The polar nanodomains are uniformly distributed in the interfacial region, which means the formation of continuous polar domains, and the corresponding PiFM spectra show the same characteristics (Figure RIII-1f). The above results provide direct evidence that polar nanodomains can be induced by the 3-D CNet at the interfacial region.

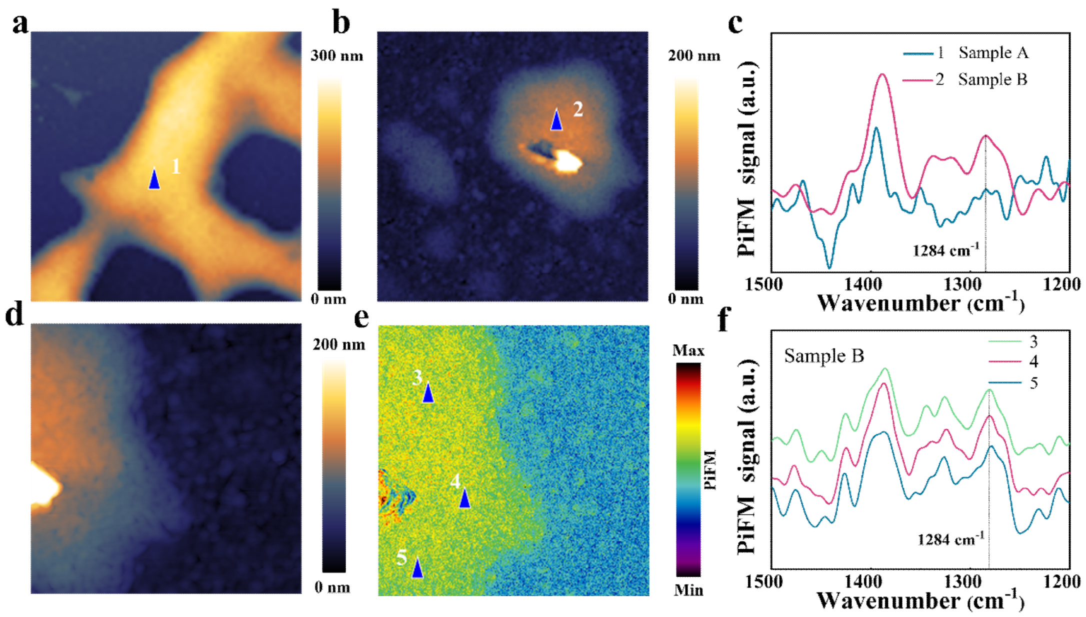

Figure RIII-1. AFM topography images (4.5 × 4.5 μm^2) of a) the sample A P(VDF-TrFE-CFE)/SiO_2/Si) and b) sample B (P(VDF-TrFE-CFE)/BCZT/SiO_2/Si). c) PiFM point spectra for two kinds of polymer samples. d) AFM height image (2 × 2 μm^2) of the sample B and corresponding PiFM image taken at 1284 cm^{-1}. f) PiFM spectra at the points marked in figure e.

Figure RIII-1 is added in **Supplementary Figure S14.**

Comment 4. One more question, volume fraction of polar phase has been analyzed in the 3-3 PCC as a function of the applied electric field. However, how to determine the specific volume fraction of polar phase? Some main equations or crucial calculation steps shall be provided in the supplementary file to make readers easier check the repeatability of the results.

Answer 4: Thank you very much for your valuable suggestions. Accordingly, we have added the relevant calculation process and formulas (Note 5. Structural origins of large electrocaloric performance). We further demonstrate that the presence of polar nanodomains in 3-3 PCC at zero field strength can significantly reduce the potential barrier for nucleation growth of polar phases. The changes in the polar phase of the neat polymer and 3-3 PCC with increasing electric field are investigated using *in situ* XRD.

The percentage of polar and non-polar phases in the crystalline volume was calculated

by the following formula:

$$f_n = \frac{A_n}{A_n + A_p} \quad \text{(Eq. S1)}$$

$$f_p = \frac{A_p}{A_n + A_p} \quad \text{(Eq. S2)}$$

The A_n is the integration areas of the non-polar phase and A_p is the integration area of the polar phase (**Supplementary Figure S12**).

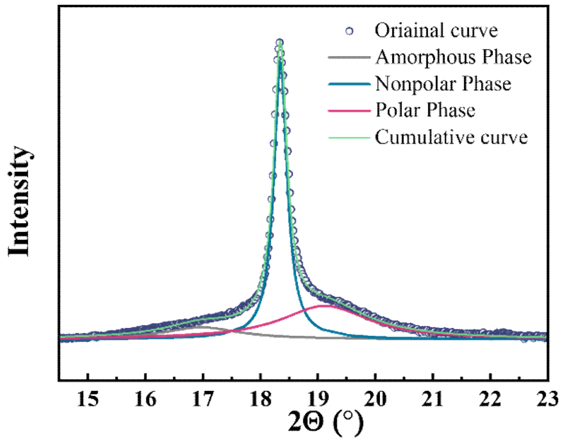

Figure RIII-2. Estimation of the fractions of the non-polar and polar phases.

Figure RIII-2 is shown in **Supplementary Figure S12.**

Comment 5. Can authors explain the reasonability of device design in this work? As a number of components including electromagnetic drive and heat flux are used in the design, thus, how to make sure the effectiveness of this device in demonstrating the electrocaloric effect performance becomes very important.

Answer 5: Thank you for pointing out this important issue. We have made a breakthrough improvement on the electrocaloric cooler, in order to make the device more effective in demonstrating the electrocaloric performance. In order to avoid mutual interference between the drive and active cooling modules, these two are effectively separated by an external 3-D printed frame. The active cooler mainly consists of electromagnet, magnetisable steel shim, heat sink, electrocaloric stack, and heat source from top to bottom (**Figure RIII-3a**). Photographs of the electrocaloric

refrigeration device that switches periodically between heat sink (top) and heat source (bottom) is shown in **Figure RIII-3b**. First, switching on the electric relay R_1, the electromagnet generates a magnetic field that attracts the steel piece at the top to move upward, while under the traction of the non-elastic tether, the electrocaloric stack at the bottom will move upward in parallel to contact the heat sink. After that, the relay R_2 is turned on. A given electric field E is applied to both sides of the electrocaloric stack. The dipoles in the electrocaloric stack are ordered in the direction of the electric field, so that the entropy inside the material decreases sharply with an increase in temperature. The heat from electrocaloric layer is transferred upward to the heat sink through the upper metal. After the heat transfer is completed, the relay R_1 is switched off and the electromagnetic field disappears. The electrocaloric stack springs back to the lower heat source under gravity and elastic force. Subsequently, relay R_2 is disconnected, and the electric field applied on electrocaloric stack is removed. The dipole in the active layer returns to the disordered state and the material entropy increases. When the temperature decreases, the electrocaloric stack absorbs heat from the lower heat source to achieve cooling (**Figure RIII-3c, d**). It is worth noting that during the whole process, the electrocaloric cooler pumps heat from the bottom heat source to the top heat sink, completing a single cycle of active cooling. To make sufficient contact between the electrocaloric stack and the cold/heat source before applying/removing the electric field to the electrocaloric cooling layer, the switching time of the relay R_2 is always 0.1 s later than that of the relay R_1 (**Figure RIII-3 e**).

Furthermore, the maximum heat flux of the electrocaloric stack on the heating and cooling side versus the applied electric field is measured by a heat flux sensor at an operation frequency of 0.1 Hz (**Figure RIII-4a**). The maximum heat fluxes for heating and cooling are 288 and -272 W m^{-2} at the electric field of 30 MV m^{-1}, respectively. **Figure RIII-4b** shows the influence of operating frequency on the average heat flux. An average cooling heat flow of -213 W m^{-2} can be achieved at a frequency of 1 Hz. Furthermore, the average cooling heat flux can be further increased at higher frequencies (-236 W m^{-2} at a frequency of 1.25 Hz). Similarly, the ideal temperature

span (1.1 K) of the electrocaloric cooling device is obtained at a frequency of 1 Hz with an electric field of 30 MV m^{-1} (**Figure RIII-4c**).

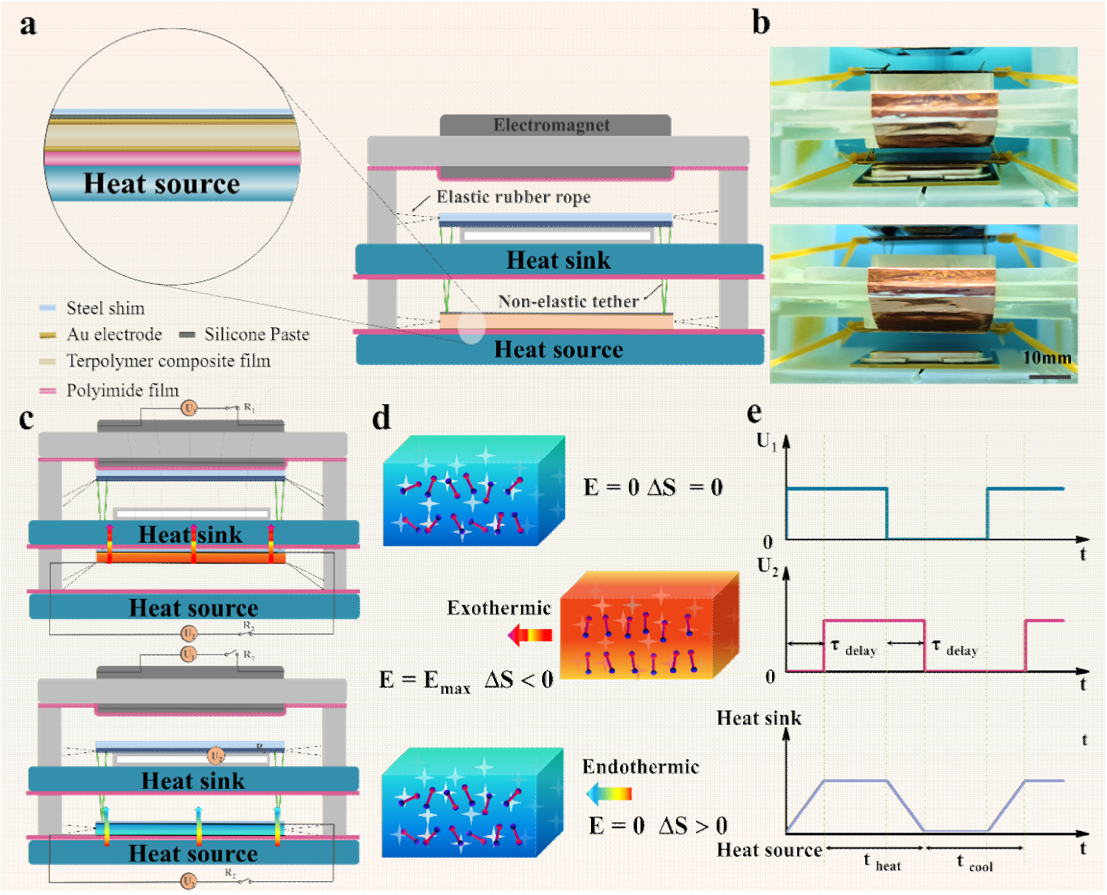

Figure RIII-3. A solid-state electrocaloric cooling device. a) Schematic illustration of the electrocaloric polymer stack and solid-state cooling device. b) Photograph of the active EC device, the elaborated main framework was obtained by 3-D printing. c) The schematic shows how an electromagnetic field drives an electrocaloric polymer stack to move heat from a heat source to a heat sink. The high-speed heat transfer from heat source to sink can be achieved by associating the active cooling of electrocaloric polymer stack with heat dissipation cycle. d) The working mechanism of the ECE based on the change of dipole entropy. e) Time domain illustration of the cooling cycle.

Figure RIII-3a-e is added in **Figure 3a-e.**

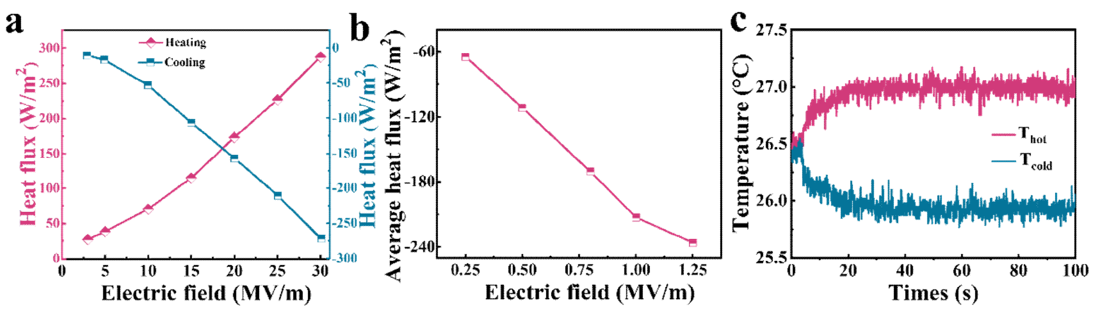

Figure RIII-4. The maximum heat flux of the electrocaloric stack on the heating and cooling side versus the applied electric field is measured by a heat flux sensor at an operation frequency of 0.1 Hz. Frequency dependence of the average heat flux at an electric field of 30 MV m^{-1}. Temperature difference of EC cooling device at an electric field of 30 MV m^{-1} and a frequency of 1 Hz.

Figure RI-5a is shown in **Figure 3f. Figures RIII-5b and c** are added in **Supplementary Figure S16.**

Comment 6. For the constructed 3D continuous network as claimed, can authors provide more information to clearly explain how to exactly achieve this kind of 3D structural design? I would like to know more details about this and then evaluate the effective/feasibility of this method. For the moment, BN is missing in the experiment part, and more details of this approach in achieving 3D network are required to guarantee the reproducibility of this method.

Answer 6: Thank you for pointing out this important issue. Embedding 3D ceramic networks into the polymer matrices and ensuring their high quality is a great challenge. In order to ensure that the continuous 3D network is successfully introduced into the polymer, the following details need to be considered. First, when filling the P(VDF-TrFE-CFE) into the 3-D CNet, it is important to repeat the process several times to ensure that the 3-D CNet is completely filled with the polymer matrix. Further, it is important to control the pressure not to be too high during the hot-pressing process. In addition, during the hot-pressing process, we place a pure film of 100 μm thickness on the top and bottom side of the hot-pressing precursor as a buffer layer. Typical thickness

after hot-pressing is around 800 μm. In order to test the electrical properties and reduce the applied voltage, the samples are thinned by abrasive paper to achieve the desired thickness.

In addition, we have added the relevant discussion from the experimental section to address this issue, with corrections as follows. *Note 1. Experimental Section: "Next, 3-3 polymer/ceramic composite (abbreviated as 3-3 PCC) materials were obtained by converting aforementioned materials to a dense body through hot-pressing at 140 °C for 10 min, under a pressure of 0.7 MPa. A 100 μm thick P(VDF-TrFE-CFE) film was placed on the top and bottom surfaces of the hot-pressed precursor as a buffer layer. The typical thickness after hot-pressing was around 800 μm. Subsequently, the samples were thinned using abrasive paper to achieve the desired thickness. Finally, the resulting 3-3 PCC films were annealed in a vacuum oven at 106 °C for 10 h to improve the crystallinity of the films".*

Comment 7. The electrocaloric cooler can only successfully function below 80 thermo degrees, otherwise high risk will emerge. Thus, I would like to know whether authors have figured out any other option or solution in improving this kind of sequence for the future work. One more serious concern is how to make sure the device only function below this certain temperature point, as it is very easy to reach 80 thermo degrees under practical circumstances.

Answer 7: Thanks for the valuable comments. The operating temperature of the electrocaloric cooling device is related to the effective working span of the ferroelectric relaxor material, i.e. P(VDF-TrFE-CFE) terpolymer. Our previous work (Appl. Phys. Lett. 2008, 92, 042903) demonstrates a ferroelectric relaxor behavior of polymer at temperature as high as 100 °C, which is much higher than that of the P(VDF-TrFE-CFE) terpolymer, i.e. near room temperature. It will be attractive for practical high-temperature electrocaloric cooler. The devices with stable operation at high temperatures are also the focus of our future work.

REVIEWERS' COMMENTS

Reviewer #1 (Remarks to the Author):

The authors have addressed the issues.

Reviewer #2 (Remarks to the Author):

The authors have finely addressed all the comments point-to-point. All the response has clearly clarified the reviewers' question. No more comments. I am glad to recommend to publish the nice work ASAP.

Reviewer #3 (Remarks to the Author):

Authors have adequately addressed most of my concern, thus, I would recommend it for publication without further revision.

Point-by-point response to the reviewers' comments:

Reviewer #1

Comments:

The authors have addressed the issues.

Answer: We are grateful to the reviewer for the thorough review and help to improve the paper.

Reviewer #2:

Comments:

The authors have finely addressed all the comments point-to-point. All the response has clearly clarified the reviewers' question. No more comments. I am glad to recommend to publish the nice work ASAP.

Answer: We appreciate the time taken for review and the positive assessment of the work.

Reviewer #3:

Comments:

Authors have adequately addressed most of my concern, thus, I would recommend it for publication without further revision.

Answer: We are grateful to the reviewer for the thorough review and help to improve the paper.
